# Neuropilin 1 (NRP1) conveys SEMA3A signals to restrict physiological angiogenesis

**DOI:** 10.1007/s10456-026-10033-z

**Published:** 2026-05-03

**Authors:** Marco Spreafico, Elena Guzzolino, Francesca Fanuele, Gaia Gestri, Carlotta Tacconi, Sara Palermo, Matilde Tricco, Valeria Catroppa, Ayazhan Aiypova, Laura Denti, Caroline Pellet-Many, Christiana Ruhrberg, Alessandro Fantin

**Affiliations:** 1 Department of Biosciences, University of Milan, Via G. Celoria 26, 20133 Milan, Italy; 2 Present Address: Institute of Clinical Physiology, National Research Council (IFC-CNR), Florence, Italy; 3 Present Address: Adaptive Immunity Laboratory, Istituto di Ricovero e Cura a Carattere Scientifico (IRCCS) Humanitas Research Hospital, Rozzano, Italy; 4 UCL Department of Cell and Developmental Biology, University College London, Gower Street, London, WC1E 6BT UK; 5 Present Address: Università Vita-Salute San Raffaele, Via Olgettina, 58, 20132 Milan, Italy; 6 Present Address: Institute of Clinical Physiology, National Research Council (IFC-CNR), Milan, Italy; 7 UCL Institute of Ophthalmology, University College London, 11-43 Bath Street, London, EC1V 9EL UK; 8 Present Address: The Francis Crick Institute, 1 Midland Rd, London, NW1 1AT UK; 9 Department of Comparative Biomedical Sciences, Royal Veterinary College, Royal College Street, London, NW1 0TU UK

**Keywords:** Neuropilin 1, SEMA3A, Angiogenesis, Zebrafish, HUVEC

## Abstract

The class 3 semaphorin SEMA3A is a secreted glycoprotein that serves as an evolutionary conserved axon repellent with proposed vascular functions. In mice, SEMA3A is dispensable for developmental brain, limb or trunk blood vessel patterning, but restricts vessel branching in the zebrafish embryo trunk. Whereas neuropilin 1 (NRP1) is thought to be the SEMA3A receptor in the mouse, prior reports identified Plexin D1 as the Sema3a receptor in zebrafish trunk vessel patterning, with previous knockdown and knockout studies yielding contradictory results on Nrp1 requirement for vessel patterning in zebrafish. To resolve these discrepancies, we have refined the prior knockdown strategy to limit off target effects and generated mutant zebrafish embryos lacking both Nrp1a and Nrp1b paralogues to show that Nrp1 restricts trunk vessel patterning in a Sema3a-dependent manner. In agreement, we found that NRP1 is required in human endothelial cells for SEMA3A-induced repulsion. Moreover, we show that SEMA3A action via NRP1 does not involve the splicing regulation of *FLT1*, previously proposed to act downstream of Plexin D1. Instead, sustained NRP1 activation independent of SEMA3A increases the expression of the anti-angiogenic soluble FLT1 (sFLT1), establishing a feedback mechanism to limit endothelial proliferation. Together, these findings demonstrate a dual role for NRP1 in shaping physiological vascular morphogenesis. Thus, NRP1 mediates repulsive SEMA3A cues in endothelial cells, in analogy to its role in axon guidance, and further restricts angiogenesis by promoting the release of sFLT1 in a SEMA3A-independent manner.

**Supplementary Information:**

The online version contains supplementary material available at 10.1007/s10456-026-10033-z.

## Introduction

Blood vessels distribute oxygen, nutrients and immune cells throughout the vertebrate body, and are a prerequisite for life in all vertebrates. During embryogenesis, a process termed angiogenesis allows blood vessels to vascularise organs as they form. In healthy adults, angiogenesis can be reactivated following injury or during disease. In both contexts, the microvasculature integrates diverse angiogenic and anti-angiogenic signals, which are sensed by endothelial cells (ECs), whereby the pro-angiogenic vascular endothelial growth factor VEGFA induces the formation of angiogenic sprouts that are led by endothelial tip cells [1, 2].

The transmembrane protein neuropilin 1 (NRP1) localises to tip cell filopodia to regulate sprouting angiogenesis [3]. As different studies, including immunoprecipitation, demonstrated that NRP1 modular extracellular domain allows interaction with both VEGFA but also the class 3 semaphorin axon guidance cue SEMA3A [4], delineating the precise angiogenic role of NRP1 in different contexts has been challenging. For example, VEGFA binding to NRP1 promotes mouse retinal angiogenesis during postnatal development but is dispensable for embryonic brain and trunk angiogenesis in the mouse [5, 6]. By contrast, exogenous SEMA3A is antiangiogenic in the chick chorioallantoic membrane assay [7] and in mouse tumour models [8, 9]. Although endothelial SEMA3A deletion was reported to impair EC tip filopodia formation in the mouse postnatal retina in an autocrine manner, this did not cause obvious vascularisation defects [10]. Moreover, endogenous SEMA3A is dispensable to regulate angiogenesis in the mouse embryonic brain, limb and trunk, and lack of semaphorin signalling via NRP1 does not affect brain developmental angiogenesis [11, 12]. Nevertheless, Sema3a was reported to prevent ectopic trunk vessel sprouting in the zebrafish embryo [13]. Zebrafish NRP1 orthologues, termed Nrp1a and Nrp1b, have not been implicated in this process, which was proposed to be mediated by Plxnd1 [13] by upregulating the soluble form of the alternative Vegfa receptor Flt1 (sFlt1, also known as sVegfr1) [14]. In contrast, in the mouse, PLXND1 serves as a SEMA3E receptor to restrict trunk vessel sprouting independently of NRP1 [15, 16], In another reported difference between mouse and zebrafish, *nrp1a* null mutants generated by genome editing nucleases were reported to lack obvious vascular defects in the trunk [17], even though other studies showed that the morpholino-mediated knockdown of either *nrp1a* or both *nrp1a* and *nrp1b* genes reduces vessel sprouting in the zebrafish trunk [18–22].

To resolve contradictory evidence about the genetic requirement for Nrp1 in zebrafish angiogenesis, we have refined and improved the previously published morpholino-based knockdown method to exclude off target effects that non-specifically disrupt trunk development and generated double mutant zebrafish for both *nrp1a* and *nrp1b*. Moreover, we complemented genetic interaction studies in zebrafish with functional assays in human ECs to demonstrate that NRP1 conveys SEMA3A signals during vascular morphogenesis.

## Results

### Nrp1a and Nrp1b knockdown causes ectopic ISV sprouting in the zebrafish embryo trunk

We and others showed that a *nrp1a* translation-blocking morpholino (MO) that partially targets *nrp1b* (*nrp1a(/b)*-MO) [22] interrupted the extension of intersomitic vessels (ISVs) in the zebrafish embryo trunk [18, 22, 23]. However, since the MO dose used in these studies was associated with toxicity [22], we sought to re-investigate the role of Nrp1a and Nrp1b using refined approaches designed to minimise off target effects. First, we generated chimeric zebrafish embryos with mosaic targeting of *nrp1a* and *nrp1b* to prevent embryo-wide MO toxicity from unspecifically affecting ISV sprouting*.* Thus, we injected *Tg(fli1a:EGFP)* embryos at 1–4 cell stage with the previously used dose of *nrp1a(/b)*-MO (0.6 pmol/embryo) versus mock controls, termed standard (Std)-MO, and then transplanted cells from these embryos into *Tg(kdrl:mCherry)* blastula-stage embryos. In contrast to prior knockdown studies that targeted *nrp1a/b* throughout the embryo, *nrp1a(/b)* knockdown ECs (EGFP+) in chimeric embryos formed ISVs that reached the dorsal side of the trunk at 36 h post fertilisation (hpf), similar to ISVs composed of host ECs (mCherry+) (Fig. S1). Moreover, *nrp1a(/b)* knockdown ECs located within ISVs extended ectopic sprouts towards the somites (Fig. S1), which are normally avascular at this developmental stage, suggesting that they had lost sensitivity to a repellent cue.

**Fig. 1:**
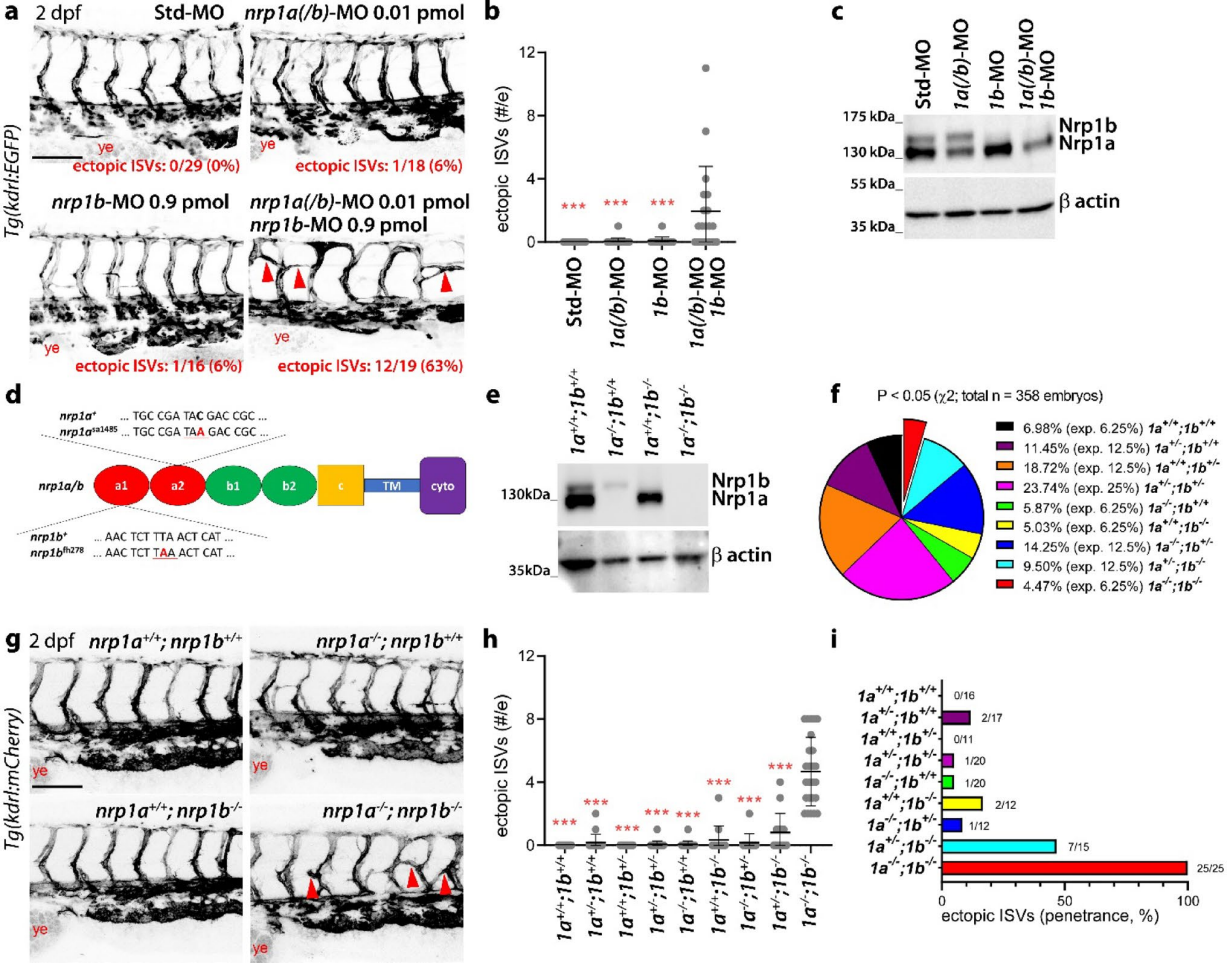
Double knockdown or knockout of Nrp1 zebrafish orthologues indicates that Nrp1a and Nrp1b prevent ectopic ISV sprouting across the somite region. **a**–**c** Analysis of *Tg(kdrl:EGFP)* zebrafish embryos that were injected at 1–4 cell stage with control standard (Std)-MO versus *nrp1a(/b)*-MO, *nrp1b*-MO and combined *nrp1a(/b)*-MO*/nrp1b*-MO at the indicated doses. **a** Representative maximum-intensity projections of confocal z stacks through embryo trunks at 2 dpf; the frequency of embryos displaying the ectopic ISV phenotype is indicated in red in each confocal image. **b** Quantification of ectopic ISVs; each data point represents the value of a single embryo (*n* ≥ 16). **c** Representative Western blotting using antibodies against C-term Nrp1 or β actin; 3 independent experiments. **d**–**i** Analysis of *Tg(kdrl:mCherry)* zebrafish embryos carrying or not nonsense mutations in both *nrp1a* and *nrp1b*. **d** Schematic representing genomic sequence and position of the *nrp1a* and *nrp1b* nonsense mutations. **e** Representative Western blotting using antibodies against C-term Nrp1 or β actin; 3 independent experiments. **f** Percentage of observed versus expected (exp.) Mendelian ratios in offsprings from double heterozygous incrosses (*P* < 0.05, Chi square test, *n* = 358 embryos). **g** Representative maximum-intensity projections of confocal z stacks through embryo trunks at 2 dpf. **h** Quantification of the number of ectopic ISVs; each data point represents the value of a single embryo (*n* ≥ 11). **i** Frequency of embryos displaying the ectopic ISV phenotype; numbers represent the frequency of embryos with phenotype over total analysed embryos per group. Red arrowheads in (**a**, **g**) indicate examples of ectopic ISVs; ye, yolk extension; scale bars: 100 µm (**a**, **g**). Graphs in (**b**, **h**) show mean ± SD; each dot represents a single embryo; e, embryo; ***, *P* < 0.001 versus combined *nrp1a(/b)*-MO*/nrp1b*-MO (**b**) or *nrp1a*^*sa1485/sa1485*^*;nrp1b*^*fh278/fh278*^ (*1a*^*−/−*^*;1b*^*−/−*^, **h**), Kruskal–Wallis test

Next, we refined the MO knockdown regimen by separately titrating *nrp1a(/b)*- and *nrp1b*-MOs for a toxicity study, in which we evaluated overall embryo morphology and scored for the presence of macroscopic anatomical defects and cardiac oedema in 2 days post fertilisation (dpf) *Tg(kdrl:EGFP)* embryos. Injecting *nrp1a(/b)*- and *nrp1b*-MOs at doses equal or below 0.025 pmol/embryo and 0.9 pmol/embryo, respectively, did not cause obvious toxicity (Fig. S2a, b). Then, we identified non-toxic doses for both MOs that did not alter ISV morphogenesis when injected singularly (subcritical doses) and co-injected them into 2 dpf *Tg(kdrl:EGFP)* embryos (Fig. 1a, all tested combinations are shown in Fig. S2c, d). We found that a combination of 0.01 pmol/embryo *nrp1a(/b)*-MO and 0.9 pmol/embryo *nrp1b*-MO efficiently reduced both Nrp1a and Nrp1b protein levels and caused the formation of ISV sprouts that ectopically crossed the somite region with high penetrance (Fig. 1a–c, Fig. S2c, d). The ectopic ISVs anastomosed in rostrocaudal direction with adjacent ISVs along the inner medial border of the somites without penetrating them, thereby forming bridge-like structures along the trunk and the tail. In summary, the double Nrp1a and Nrp1b knockdown prevented ISV repulsion from the somite region, consistent with the chimeric embryo results.

### Genetic Nrp1a and Nrp1b targeting causes ectopic ISV sprouting in the zebrafish embryo trunk

In a third approach, we generated double mutant zebrafish embryos lacking both Nrp1 paralogues by combining the *nrp1a*^*sa1485*^ [24, 25] and *nrp1b*^*fh278*^ [26] mutant lines, each carrying a premature termination codon within the Nrp1 a2 and a1 domain, respectively (Fig. 1d). Western blot analysis using an antibody specific for the Nrp1 C-terminal domain confirmed lack of full length Nrp1a and Nrp1b proteins in 5 dpf double mutants (Fig. 1e). When generated from a double heterozygous incross, double mutants were present at a slightly lower frequency than expected (Fig. 1f), suggesting that loss of both Nrp1a and Nrp1b causes a small fitness reduction. Nevertheless, surviving double mutants reached adulthood and were fertile. Similar to MO-mediated knockdown, the simultaneous loss of Nrp1a and Nrp1b in the *Tg(kdrl:mCherry)* background resulted in the formation of ectopic ISV sprouts at 2 dpf (Fig. 1g, h) with complete penetrance (Fig. 1i).

**Fig. 2:**
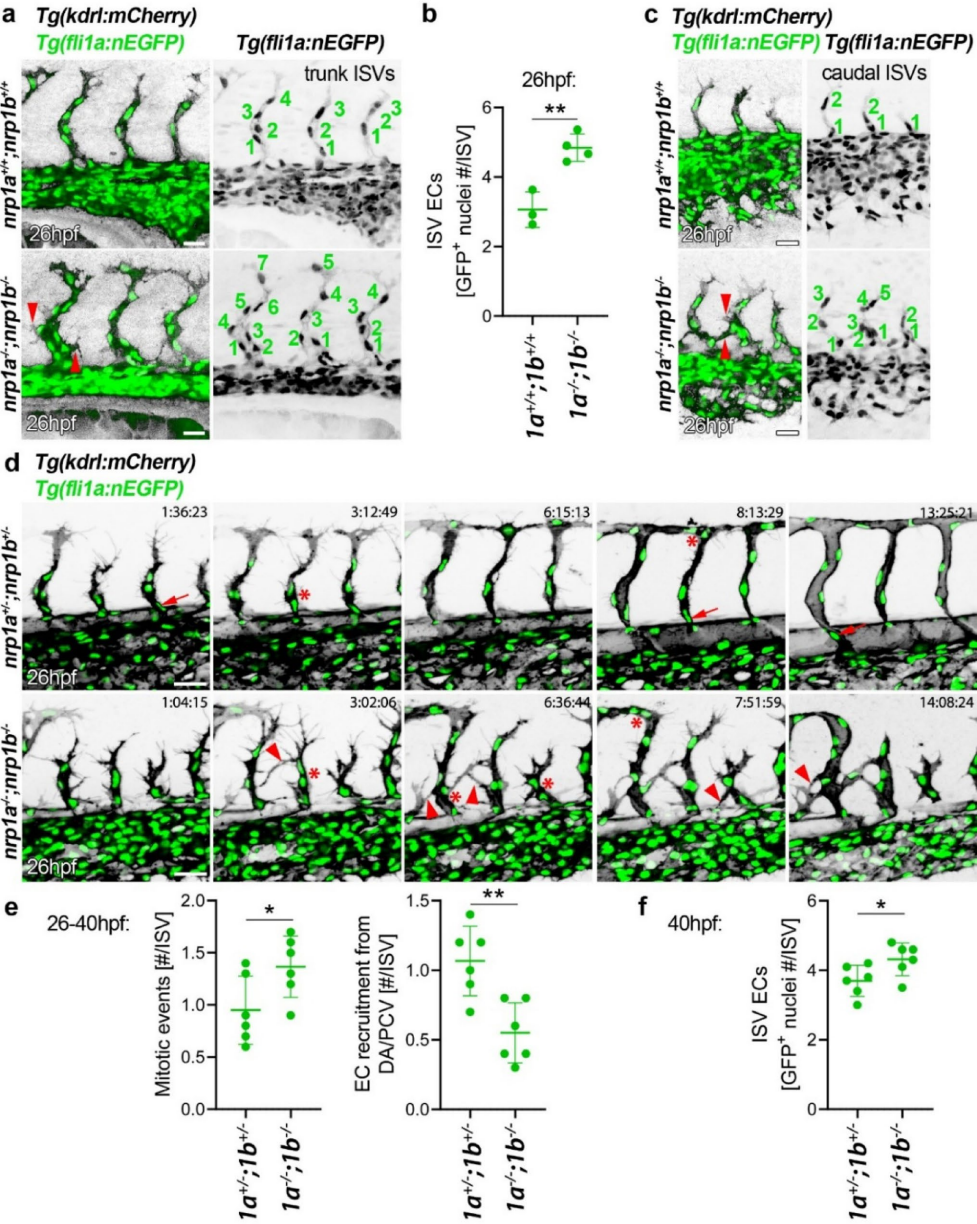
Lack of Nrp1 results in increased EC proliferation within each ISV. **a**–**f** Trunk vascularisation analysis of *Tg(kdrl:mCherry);Tg(fli1a:nEGFP)* zebrafish embryos carrying or not nonsense mutations in both *nrp1a* and *nrp1b.* **a**–**c** Representative maximum-intensity projections of confocal z stacks at 26 hpf through the central part of the trunk (**a)** corresponding quantification of EC number in all fully extended ISVs in each embryo trunk in (**b**); each data point represents the value of a single embryo, *n* ≥ 3) and the caudal part of the trunk (**c**); numbers indicate the different EC nuclei in each ISV. **d** Representative maximum-intensity projections of confocal z stacks at the indicated time points starting from 26 hpf; time display refers to hours:minutes:seconds. **e**, **f** Quantification of mitotic events and EC recruitment from either DA or PCV (**e**) and EC number (**f**) per fully extended ISV; each data point represents the value of a single embryo (*n* = 6). In (**a**, **c**, **d**), red arrowheads, asterisks and arrows indicate examples of ectopic ISVs, EC mitosis and EC recruitment, respectively; scale bars: 20 µm (**a**, **c**), 50 µm (**d**). Graphs in (**b**, **e**, **f**) show mean ± SD; *, *P* < 0.05; **, *P* < 0.01, unpaired Student-t test

ISVs are first formed by primary sprouting from the dorsal aorta (DA) between 22 and 36 hpf followed by secondary sprouting from the posterior cardinal vein (PCV) at 32–48 hpf. Therefore, we evaluated if ectopic ISV sprouts are caused by defects in primary ISV sprouting. At 26 hpf, primary ISVs in both double *nrp1a* and *nrp1b* morphants and mutants elongated towards the dorsal side of the trunk, as seen in controls; however, in contrast to controls, they extended filopodia-studded vessel sprouts across the somite region in both central and caudal trunk regions (Figs. 2a–c, S3a, b, Movies M1, 2). Using the endothelial nuclear reporter line *Tg(fli1a:nEGFP)* alongside *Tg(kdrl:mCherry)*, we found that ISVs in double morphants and double mutants were composed of a significantly higher number of ECs (Figs. 2a–c, S3a–c). This increased EC number was already observed before ectopic sprouts elongated towards the somite region and in newly generated sprouts that had not yet reached the dorsal trunk (Figs. 2a–c, S3a–c). As ECs positive for the mitotic marker phosphorylated histone H3 (pHH3) were too rare in a single time snapshot at 26 hpf for quantitative scoring (Fig. S3a–c), we instead investigated EC proliferation by scoring EC mitotic events in each ISV via time lapse analysis (Fig. 2d, e, Movie M3). Compared to controls, double mutants showed a significantly increased number of EC mitotic events (Fig. 2d, e, Movie M4). Although this was accompanied by reduced EC recruitment from either the dorsal aorta or posterior cardinal vein, double mutant ISVs still contained more ECs by 40 hpf than control ISVs (Fig. 2f). These findings suggest that ectopic ISV sprouting across the somite region in double morphant or mutant embryos results from lack of EC repulsion combined with increased EC proliferation within each primary ISV, feeding the ectopic ISV sprouts.

### Sema3a expression during ISV sprouting in the zebrafish embryo trunk

**Fig. 3:**
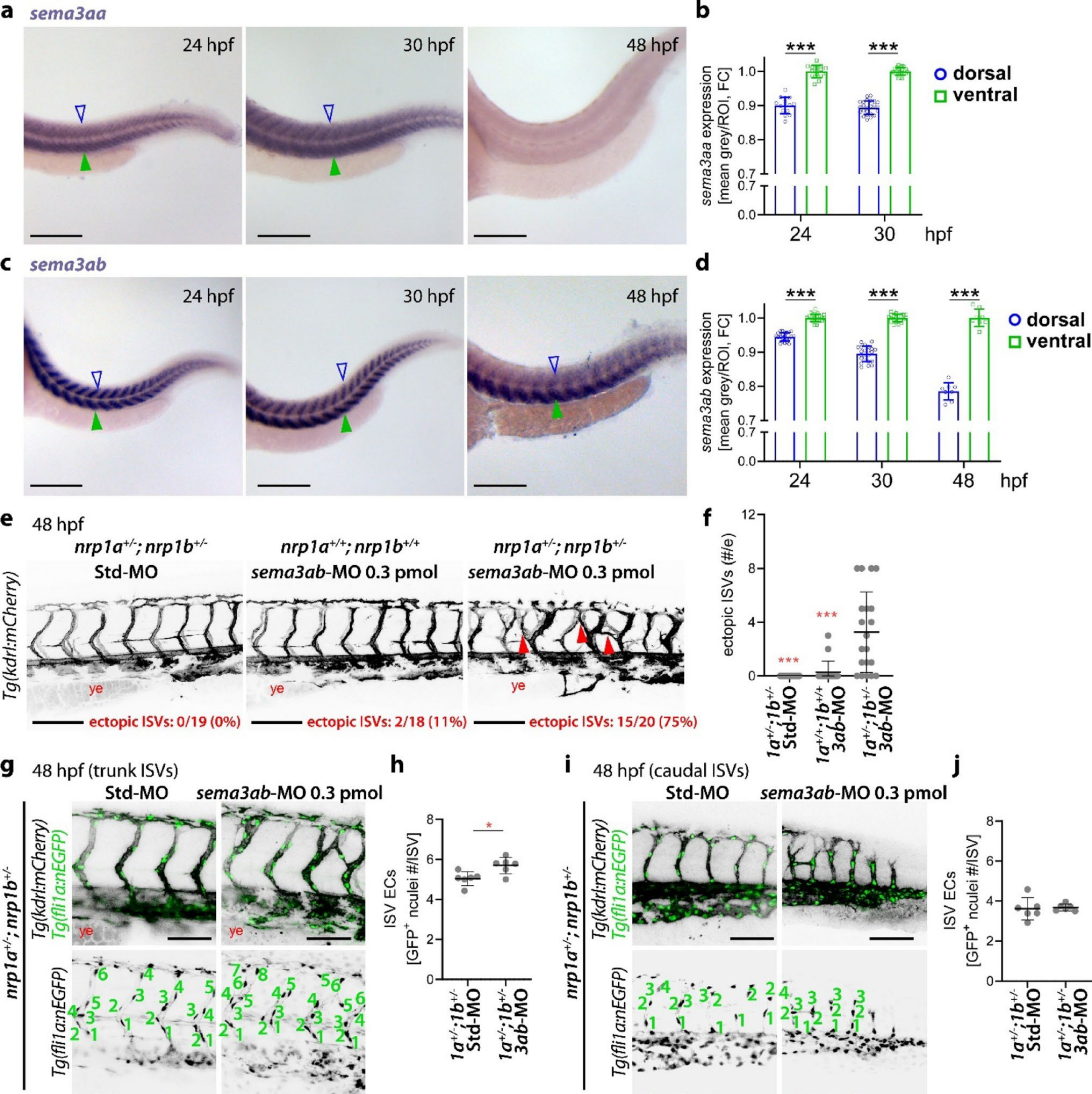
Nrp1 and Sema3a genetically interact to prevent ectopic ISV sprouting. **a**–**d** Time course analysis of Sema3a expression in zebrafish embryos during trunk vascularisation. **a**, **c** Representative pictures of whole mount in situ hybridisation using antisense probes for *sema3aa* (**a**) and *sema3ab* (**c**); green full arrowheads and blue contour arrowheads indicate expression of transcripts for Sema3a ligands in the ventral and dorsal somites, respectively. **b**, **d** In situ hybridisation signal quantification in both ventral and dorsal portion of somites for *sema3aa* (**b**, *n* ≥ 15) and *sema3ab* (**d**, *n* ≥ 8); each data point represents the value of a single embryo. **e**, **f** Trunk vascularisation analysis of *Tg(kdrl:mCherry)* zebrafish embryos carrying or not nonsense heterozygous mutations in both *nrp1a* and *nrp1b* that were injected at 1–4 cell stage with control standard (Std)-MO or *sema3ab*-MO at the indicated doses. **e** Representative maximum-intensity projections of confocal z stacks through embryo trunks at 2 dpf; the frequency of embryos displaying the ectopic ISV phenotype is indicated in red in each confocal image; red arrowheads indicate examples of ectopic ISVs; ye, yolk extension. **f** Quantification of ectopic ISVs; each data point represents the value of a single embryo (*n* ≥ 18). **g**–**j** Trunk vascularisation analysis of *Tg(kdrl:mCherry);Tg(fli1a:nEGFP)* zebrafish embryos carrying nonsense mutations in both *nrp1a* and *nrp1b* that were injected at 1–4 cell stage with control standard (Std)-MO or *sema3ab*-MO at the indicated doses*.* **g**, **i** Representative maximum-intensity projections of confocal z stacks at 48 hpf through the central (**g**) and caudal (**i**) parts of the trunk. **h**, **j** Corresponding quantification of EC number in all fully extended ISVs in each embryo trunk (**h**) and caudal (**j**) region; each data point represents the value of a single embryo, *n* = 6); numbers indicate the different EC nuclei in each ISV. Scale bars: 250 µm in (**a**, **c**); 100 µm in (**e**, **g**, **i**). In (**b**, **d**, **f**, **h**, **j**), graphs show mean ± SD; *, *P* < 0.05; ***, *P* < 0.001; 2-way ANOVA followed by Sidak’s multiple comparison’s test (**b**, **d**), Kruskal–Wallis test (versus *1a*^*+/−*^*;1b*^*+/−*^ *3ab*-MO injection,** f**) or unpaired Student-t test (**h**, **j**)

The above complementary loss-of-function strategies all showed that the ISV expansion and ectopic sprouting caused by the combined loss of Nrp1a and Nrp1b was similar to the previously described phenotype caused by loss of Sema3ab [13]. Moreover, both Sema3a paralogue genes, *sema3aa* and *sema3ab*, are expressed in the somites between 15 and 24 hpf [13, 27–29]. To better understand the relationship of both Sema3a paralogues to ISV morphogenesis, we performed whole mount in situ hybridisation for *sema3aa* and *sema3ab* between 24 and 48 hpf, when ectopic ISVs form with Nrp1 or Sema3ab deficiency. Both paralogues were expressed in the dorsal and ventral halves of the somites, with the *sema3aa* signal appearing more diffuse and becoming barely detectable at 48 hpf, whereas the *sema3ab* signal showed a somite-specific pattern throughout the entire mediolateral extension of the somites at all stages examined (Figs. 3a–d, S4a, b). The expression of both paralogues appeared significantly more abundant in the ventral than dorsal somites (Figs. 3a–d, S4a, b). In particular, *Sema3ab*, whose knockdown caused ectopic ISV sprouting (Fig. S4c, d), as previously reported [13], showed a more defined expression pattern that persisted in the somites throughout the ISV morphogenesis window. These expression patterns are consistent with Sema3aa and Sema3ab providing chemorepulsive cues that prevent Nrp1-expressing ISVs from sprouting across the somites.

### Nrp1 and Sema3a cooperate to prevent ectopic ISV sprouting independently of sFlt1

To evaluate whether Nrp1 and Sema3a genetically interact, we focussed on the Sema3ab paralogue because it showed a stronger and more persistent expression pattern in the somites than Sema3aa (Figs. 3a–d, S4a, b). We first determined a subcritical dose for *sema3ab*-MO that did not cause ectopic ISVs or other obvious morphological defects. In wild type embryos, a single dose of 0.6 pmol/embryo of *sema3ab*-MO caused ectopic ISV sprouting (Fig. S4c, d), whereas the lower 0.3 pmol/embryo dose did not (Figs. S4e, 3e, f). In double *nrp1a* and *nrp1b* heterozygous mutants, injecting the subcritical dose of 0.3 pmol/embryo of *sema3ab*-MO did instead cause ectopic ISVs with 75% penetrance (Fig. 3e, f), which was not the case in untreated (Figs. 1h, 2d) or after Std-MO injection (Fig. 3e, f). Similarly, co-injection of 0.3 pmol/embryo of *sema3ab*-MO with the subcritical combination of 0.01 pmol/embryo of *nrp1a(/b)*-MO and 0.1 pmol/embryo of *nrp1b*-MO caused significant ectopic ISVs (72% penetrance; Fig. S4e, f). Hence, both our complementary strategies indicate that Nrp1 cooperates with Sema3a to prevent vascular overgrowth in the zebrafish embryo trunk.

**Fig. 4:**
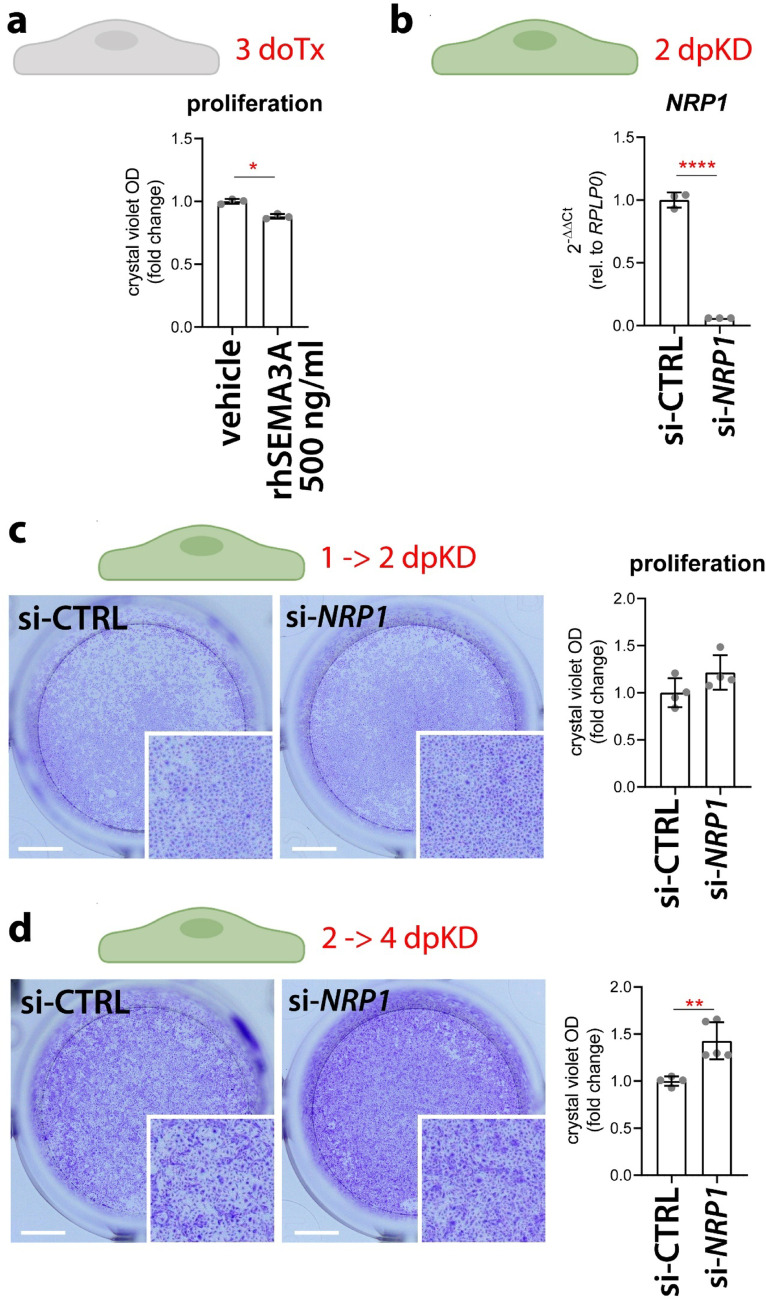
NRP1 and SEMA3A regulate human EC proliferation. **a** Quantification of proliferation assays on HUVECs treated with vehicle or 500 ng/ml recombinant human SEMA3A (rhSEMA3A) over 3 days of treatment (doTx). Each data point represents the average value from one independent experiment (*n* = 3 independent experiments); paired Student-t test. **b** RT-qPCR expression analysis of *NRP1* transcripts normalized to *RPLP0* in HUVECs at 2 days post knockdown (dpKD) with scramble siRNA or siRNA targeting *NRP1*. Each data point represents the value of a single well from one representative experiment out of 2 independent experiments (*n* = 3 wells); unpaired Student-t test. **c**, **d** Quantification of proliferation assays on HUVECs transfected with scramble siRNA or siRNA targeting *NRP1* between 1 and 2 (**c**) or 2 and 4 dpKD (**d**). Each data point represents the value of a single well from one representative experiment out of 2 independent experiments with 2 different batches of HUVECs (*n* ≥ 4 wells); unpaired Student-t test. Expression levels are shown as mean ± SD; *, *P* < 0.05; **, *P* < 0.01; ****, *P* < 0.0001

When double heterozygous mutants were injected with the subcritical dose of *sema3ab*-MO (0.3 pmol), we found that the ISVs in the 2 dpf embryo trunk, where ectopic ISV were detectable, were composed by a higher number of ECs than double heterozygous mutants injected with control Std-MO (Fig. 3g, h). However, we did not observe any difference in the number of ECs in the newest ISV sprouts in the caudal part of the trunk (Fig. 3i, j), which were instead characterised by increased EC number in the double *nrp1a* and *nrp1b* homozygous mutants (Fig. 2c). In agreement with this observations, stimulation of cultured human umbilical vein ECs (HUVECs) with recombinant, furin-activated human SEMA3A induced a small but significant reduction in cell proliferation over 3 days (Fig. 4a); moreover, NRP1 silencing in HUVECs (Fig. 4b) via a previously validated siRNA [30] did not affect proliferation at 2 days post knockdown (dpKD, Fig. 4c), but at 4 dpKD consistently led to a significant increase in their proliferation rate (Fig. 4d), consistent with the double *nrp1a* and *nrp1b* mutant phenotype (Fig. 2).

**Fig. 5:**
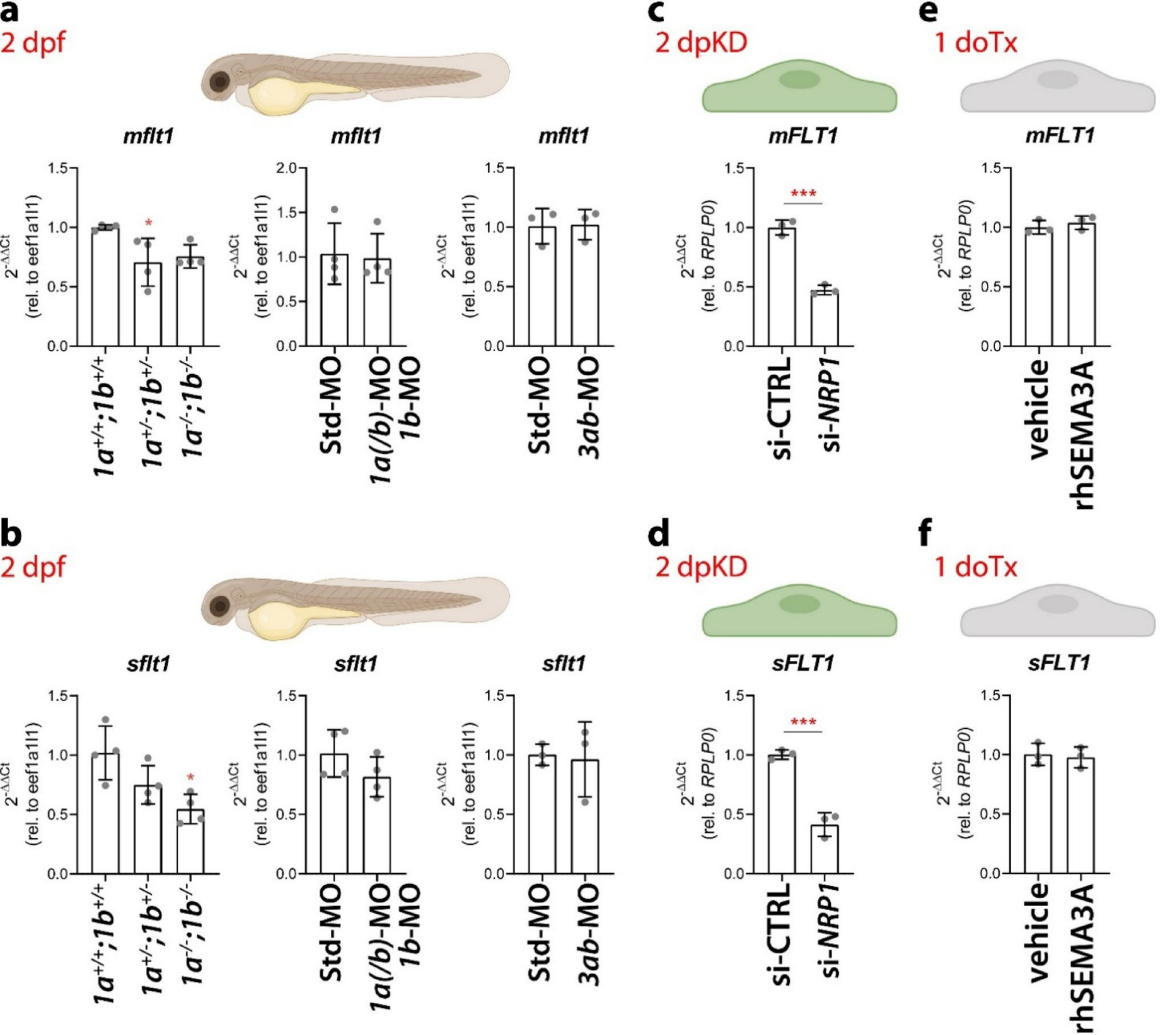
NRP1 and SEMA3A differentially regulate transcript abundance of mFLT1 and sFLT1. **a**, **b** RT-qPCR expression analysis of transcripts for membrane-bound (*mflt1,* **a**) and soluble Flt1 (*sflt1,* **b**), normalized to *eef1a1l1* in 2 dpf zebrafish embryos carrying or not nonsense mutations in both *nrp1a* and *nrp1b* (left graphs), or injected at 1–4 cell stage with Std-MO versus combined *nrp1a(/b)*-MO (0.01 pmol/embryo)*/nrp1b*-MO (0.9 pmol/embryo) (centre graphs) or *sema3ab*-MO (0.6 pmol/embryo) (right graphs). Each data point represents the value of a pool of 20 embryos from one independent experiment (*n* ≥ 3 independent experiments); one-way ANOVA followed by Tukey’s multiple comparisons test (left graphs); paired Student-t test (centre and right graphs). **c**–**f** RT-qPCR expression analysis of transcripts for membrane-bound (*mFLT1*, **c**, **e**) and soluble FLT1 (*sFLT1,* **d**, **f**), normalized to *RPLP0* in HUVECs transfected with scramble siRNA or siRNA targeting *NRP1* (**c**, **d**), or HUVECs exposed to SEMA3A for 24 h (**e**, **f**); dpf, days post fertilisation; dpKD, days post knockdown; doTx, days of treatment. Each data point in (**c**, **d**) represents the value of a single well from one representative experiment out of 2 independent experiments with 2 different batches of HUVECs (*n* = 3 wells); unpaired Student-t test. Each data point in (**e**, **f**) represents the average value from one independent experiment (*n* = 3 independent experiments); paired Student-t test. Expression levels are shown as mean ± SD; *, *P* < 0.05; ***, *P* < 0.001

Sema3ab was previously hypothesised to restrict vascular sprouting in the zebrafish embryo trunk by signalling via Plxnd1 [13], which in turn inhibits vascular expansion by promoting the expression of the VEGFA trap sFlt1 via alternative splicing of the *flt1* gene [14]. Therefore, we measured transcript levels of the membrane-bound (*mflt1*) and soluble (*sflt1*) *flt1* alternative splicing isoforms in the trunk of 2 dpf mutant embryos or embryos injected with either the combination of *nrp1a(/b)-*MO (0.01 pmol/embryo) and *nrp1b*-MO (0.9 pmol/embryo) or the single *sema3ab*-MO (0.6 pmol/embryo) that induced ectopic ISVs (Fig. 1a, b, Fig. S4c, d). RT-qPCR analysis using genomic DNA-resistant primers showed that the levels of both *sflt1* and *mflt1* transcripts in *sema3ab* morphants were not affected (Fig. 5a, b). In contrast, loss of both Nrp1 paralogues in double *nrp1a* and *nrp1b* mutants resulted in a dose-dependent decrease in *sflt1* mRNA, with the lowest and significantly different levels in homozygous mutants (Fig. 5b), whereas double heterozygous mutants showed an intermediate phenotype, with a non-significant trend for reduced levels (Fig. 5b). Combined knockdown of the Nrp1 paralogues, which reduced Nrp1a and Nrp1b protein levels without fully depleting them (Fig. 1c), similarly caused a trend toward reduced *sflt1* expression, comparable to the loss of one allele of each single gene in the double heterozygotes (Fig. 5b). *mflt1* transcript levels showed similar dynamics to the *sflt1* in both *nrp1a*;*nrp1b* morphants and mutants, although the reduction in double mutants was less pronounced and not significant (Fig. 5a). Consistent with our zebrafish data, HUVECs depleted for NRP1 also showed a significant reduction in transcripts for sFLT1 (and mFLT1) at 2 dpKD (Fig. 5c, d), whereas overnight stimulation of human ECs with recombinant, furin-activated human SEMA3A did not alter *sFLT1* or *mFLT1* mRNA levels (Fig. 5e, f).

Taken together with our genetic interaction experiments (Figs. 3, S4), these results support a model in which Sema3ab interacts with Nrp1 to repel ISVs from the somite region through a mechanism independent of Flt1 regulation. Nevertheless, Nrp1 may promote sFlt1 expression to restrict EC proliferation that is known to be induced by sFLT1 target, namely VEGFA.

### NRP1 mediates SEMA3A repulsive cues in human ECs

**Fig. 6:**
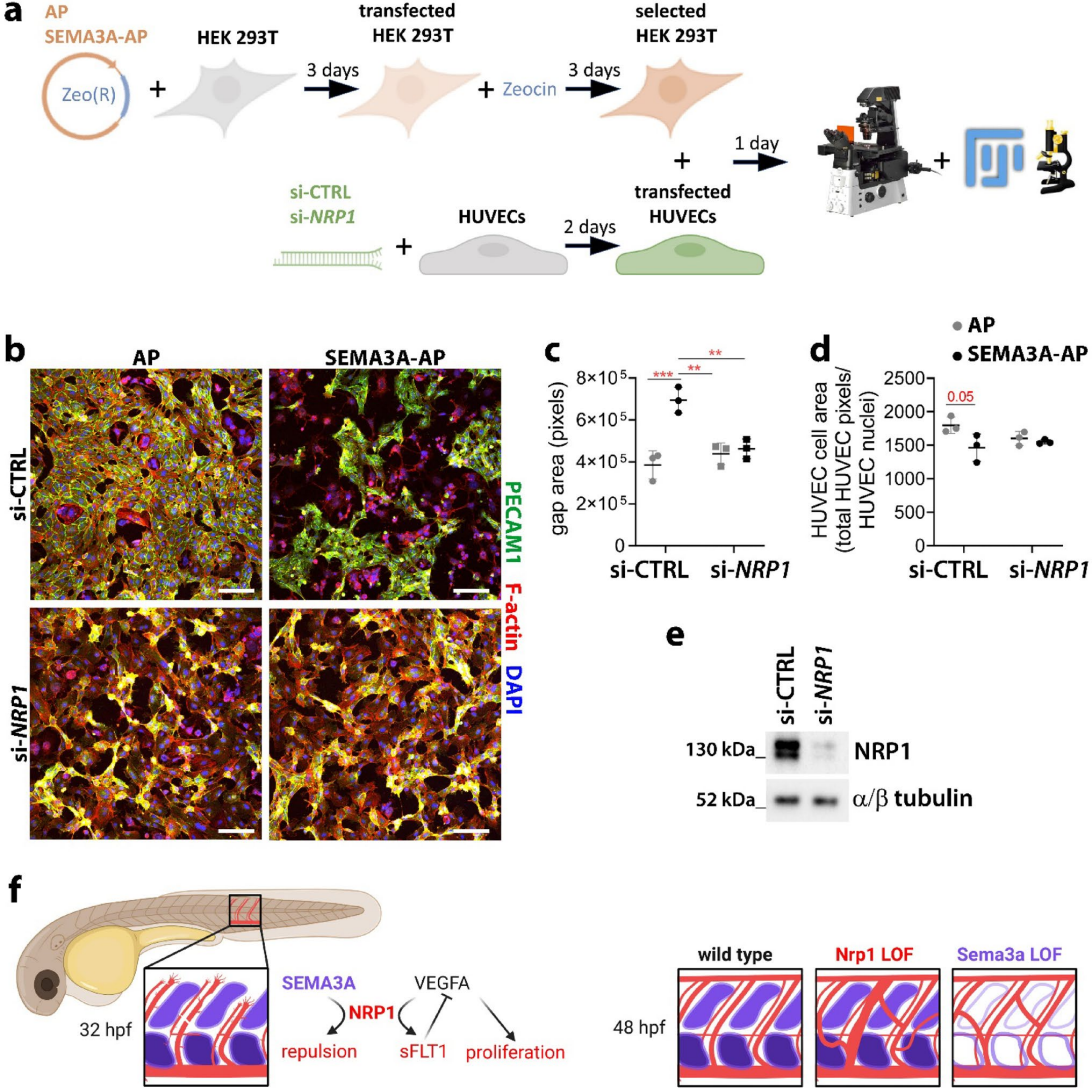
NRP1 is required to mediate SEMA3A repulsive cues in ECs. **a**–**e** In vitro repulsion assay by coculturing HUVECs with AP (alkaline phosphatase) only or SEMA3A-AP expressing HEK 293 T cells. **a** Schematic depicting the experimental strategy for the EC repulsion assay. **b** Representative maximum-intensity projections of confocal z stacks through HUVECs/HEK 293 T cocultures; scale bars: 100 µm. **c**, **d** Quantification of gap areas in the EC monolayer (**c**) and HUVEC single cell area (**d**); graphs show mean ± SD; each data point represents the average of 3 wells for each independent experiment; *n* = 3 independent experiments; **, *P* < 0.01; ***, *P* < 0.001, 2-way ANOVA followed by Tukey’s multiple comparisons test. **e** Representative Western blotting using antibodies against NRP1 or α/β tubulin on lysates from HUVECs transfected with scramble siRNA or siRNA targeting *NRP1*. **f** Proposed working model, whereby somite-derived SEMA3A acts through NRP1 on ISV ECs to repel ECs from the somite region and, concomitantly but independent of SEMA3A, NRP1 can increase sFLT1 levels, which in turn inhibit VEGFA from inducing EC proliferation (left). By 48 hpf, both Nrp1 and Sema3a loss of function (LOF) strategies result in ectopic ISVs crossing the somite region (right). Created with BioRender.com

To investigate the cellular and molecular mechanisms by which SEMA3A and NRP1 cooperate to shape vascular morphogenesis, we co-cultured SEMA3A-expressing human embryonic kidney (HEK) 293 T cells with HUVECs (Fig. 6a). When mixed with mock transfected HEK cells, HUVECs formed a dense monolayer (Fig. 6b). In contrast, co-culture with SEMA3A-expressing HEK cells resulted in HUVEC-free areas surrounding HEK cells and a reduced HUVEC single cell area (Figs. 6b–d, S5a, b). Tracking mitotic events with the cell-permeant nuclear marker Hoechst 33342 in green fluorescent protein (GFP)-expressing HUVECs co-cultured with either mock or SEMA3A-expressing HEK cells did not reveal any differences in proliferation in either HUVECs or HEK cells (Fig. S5c, d). Thus, SEMA3A-induced repulsion in our co-culture system occurs independently of proliferation changes (Movies M5, M6). In agreement with our in vivo data, NRP1 knockdown in HUVECs (Fig. 6e) significantly suppressed SEMA3A ability to repel ECs (Fig. 6b–d). These findings demonstrate that NRP1 mediates SEMA3A chemorepulsive cues cell autonomously in ECs (Fig. 6f).

## Discussion

Our results suggest that the main role of Nrp1 during zebrafish trunk vascularisation is to mediate signals that restrict lateral blood vessel sprouting (see working model in Fig. 6f). These findings are based on the observations that double knockdown or knockout of Nrp1 zebrafish orthologues resulted in lack of ISV repulsion from the somite region (Fig. 1) and consequent ISV overgrowth (Fig. 2). This observation contrasts past studies using either a knockdown or a knockout strategy in zebrafish. In particular, prior studies using a MO-mediated knockdown of either Nrp1a or both Nrp1a and Nrp1b reported defective ISV extension towards the dorsal larval trunk [18–22]. As we had found that the dose most often employed in previous studies for the translation-blocking MO targeting both *nrp1a* and *nrp1b* was associated with general toxicity that prevented proper embryo development [22], we have here refined the Nrp1 knockdown approach to avoid off target effects: chimeric embryos with mosaic knockdown of *nrp1a* and *nrp1b* (Fig. S1) and combining subcritical doses of 2 different MOs (Figs. 1, S2). In both cases, ISV elongation towards the dorsal side of the trunk was not affected, whereas we observed ectopic ISV extension across the somite region (Figs. 1, S1, S2). Nrp1 knockout strategies in zebrafish have to date been limited to Nrp1a, without reported vascular defects [17], except slightly impaired collective EC migration in the common cardinal vein [31]. Consistent with these prior studies and with both Nrp1 paralogues being expressed in the ISVs with a similar spatiotemporal pattern [18, 19, 25], we found that both Nrp1a and Nrp1b were each individually dispensable for ISV formation. However, consistent with the refined double MO knockdown strategy, the simultaneous loss of both paralogues in double mutants resulted in ISVs with normal dorsal extension but ectopic invasion of the somite region (Fig. 1). Transcriptional adaptation is a recently described genetic compensation by which related gene(s) are upregulated downstream of mutant mRNA degradation [32]. However, the redundant requirement for each single Nrp1 paralogue is unlikely due to transcriptional adaptation, because the *nrp1a* mutation employed in our study did not increase Nrp1b expression (Fig. 1) [25].

The only reported vascular defects for Nrp1a loss was slightly impaired Sema3d-induced collective EC migration in the common cardinal vein [31]. Consistent with an additional role for Nrp1 in mediating semaphorin signalling also in trunk ISVs, we have observed ectopic sprouting in both *nrp1a* and *nrp1b* double mutants and morphants at a developmental stage when the trunk region expresses the SEMA3A orthologues Sema3aa and Sema3ab (Figs. 3, S4). In agreement with a role for NRP1 as a SEMA3A receptor, genetic interaction experiments in zebrafish showed that Nrp1a and Nrp1b prevent ectopic ISV sprouting in the somite region by cooperating with Sema3ab (Figs. 3, S4), the Sema3a paralogue previously implicated with the modulation of vascular repulsion in zebrafish embryos [13]. Ectopic ISVs observed with the *sema3ab*-MO critical dose, with the *sema3ab*-MOs subcritical dose injected into *nrp1a* and *nrp1b* double heterozygous mutants or with the triple combination of *nrp1a(/b)*-*, nrp1b*- and *sema3ab*-MOs subcritical doses were most frequent across the dorsal portion of the somite (Figs. 3, S4), which may be explained by the most effective loss of Sema3ab in knockdown experiments in those regions that are less abundant in *sema3ab* transcripts compared to the ventral half of the somites (Figs. 3, S4).

Rather than a predominant pro-angiogenic effect, as observed in the brain and retina of mice [3, 5, 22, 30, 33, 34], we found that the main role of Nrp1 during trunk vascularisation in the zebrafish is to mediate SEMA3A signals that restrict blood vessel sprouting (Figs. 1, 3). Interestingly, this finding differs from findings in mouse embryos, in which SEMA3A is dispensable for trunk vascular patterning [11]. The discrepancy might be due to a more restricted and superficial SEMA3A expression in mouse embryonic somites [35] than in zebrafish, whereby transcripts for Sema3a accumulated throughout the mediolateral extension of the somites (Fig. S4). SEMA3A or semaphorin signalling via NRP1 were also shown to be dispensable for vascularisation of the mouse embryonic hindbrain [11], where SEMA3A is expressed at low levels [36] when compared to the strong and highly stereotyped expression of SEMA3A orthologues in the zebrafish trunk (Figs. 3, S4). Importantly, in support of our zebrafish observations, we found that SEMA3A repelled human ECs via NRP1 (Fig. 6), in accordance with a previous report demonstrating that SEMA3A reduced ECs migration towards extracellular matrix cues in a NRP1-dependent fashion [37]. Moreover, our human co-culture assay showed that expression of NRP1 specifically in ECs mediates SEMA3A repulsive signals (Fig. 6). Such EC autonomous role for NRP1 in negatively regulating angiogenesis has been previously hypothesised to be partly complemented in pathological settings by an effect of SEMA3A on recruitment of NRP1-expressing monocytes [38].

SEMA3A has previously been reported to inhibit EC proliferation during mouse kidney development [39] and in cultured human ECs [40–42]. In agreement, HUVEC exposure to SEMA3A led to decreased EC proliferation (Fig. 4) and Sema3ab knockdown in combination with Nrp1 (Fig. 3) resulted in ISVs with a higher number of ECs, even though only in the trunk region where ectopic sprouts occur (Fig. 3). These observations suggest that the ectopic sprouts induced by lack of Sema3a-mediated repulsion via Nrp1 are also associated with an increase in EC proliferation to support the extension of the unrestricted vessel sprouts. Interestingly, the lack of Nrp1 in double mutant or morphant zebrafish embryos resulted in a widespread increase in EC proliferation in every ISV, including the ones that are not mispatterned and overgrown yet (Figs. 2, S3). Our in vivo observations were replicated in vitro in NRP1-silenced ECs, but only after 4 days from NRP1 knockdown (Fig. 4), suggesting that NRP1-mediated repression of proliferation occurs after sustained NRP1 activation. Further investigation might address whether the increase in EC proliferation in double Nrp1 mutant or morphant zebrafish embryos may also be accompanied by raised levels of proangiogenic growth factors, such as VEGFA.

Sema3a was previously suggested to promote ISV repulsion in zebrafish by binding to Plxnd1, which in turn induces upregulation of sFlt1 [14]. However, we found that mRNA transcripts for sFlt1 were not altered by Sema3ab loss of function in zebrafish embryos or by SEMA3A administration to cultured human ECs (Fig. 5). Our results therefore indicate that SEMA3A might engage in a complex with a plexin family member different from PLXND1. For example, PLXNA1 was previously shown to mediate SEMA3A inhibition of human EC migration towards extracellular matrix [37] and SEMA3A signals in lymphatic ECs for lymphatic valve morphogenesis [43]. Moreover, we recently demonstrated that PLXNA2 is the most abundantly expressed class A plexin in both human and mouse ECs [44]. Even though it is still possible that PLXND1 activation regulates sFLT1 expression, our data agree with lack of defects in ISV primary (and secondary) sprouting in zebrafish embryos lacking Flt1 [45, 46], further supporting that the EC repulsion induced by SEMA3A-NRP1 interaction is independent from the release of sFLT1.

In contrast to Sema3a knockdown and HUVEC treatment with SEMA3A, loss of both Nrp1 paralogues in zebrafish embryos and NRP1-silencing in HUVECs showed that NRP1 is required to promote the expression of the antiangiogenic, VEGFA-decoy receptor sFLT1 (Fig. 5), in agreement with a recent report showing reduced sFLT1 levels in HUVECs following treatment with the NRP1-specific inhibitor EG00229 [47]. Interestingly, sFLT1 mRNA and protein levels have been demonstrated to increase following systemic VEGFA overexpression in mice as well as HUVEC treatment with VEGFA, with raised sFLT1 levels resulting in reduced EC proliferation [48]. Our data demonstrating that sustained NRP1 activation results in sFLT1 upregulation and reduced proliferation in ECs, are thus compatible with an EC autonomous, negative feedback mechanism to limit VEGFA-proangiogenic effects. A VEGFA-sFLT1 negative feedback loop, whereby VEGF triggers the production of sFLT1, which in turn binds to and neutralizes VEGF, has previously been demonstrated in preeclampsia studies and suggested to contribute to pregnancy complications [49]. Thus, our results indicate that NRP1 might play a hitherto unidentified key role in this clinically relevant process.

In addition to class 3 semaphorins, NRP1 modular extracellular domain allows interaction with other ligands, such as VEGFA, with Vegfa signalling in zebrafish being essential to promote the sprouting and elongation of ISVs [50–52]. However, Nrp1 loss in our refined knockdown and knockout strategies did not reduce ISV elongation towards the dorsal side of the trunk (Fig. 1). Even though different studies reported NRP1 as a positive regulator of blood vessel morphogenesis in mouse [3, 12, 22, 30], a limited role for Nrp1 in Vegfa signalling in zebrafish still agrees with previous observations made in mouse embryos, whereby mutants lacking VEGFA binding to NRP1 do not show major vascular defects [5, 6].

In conclusion, our results resolve previous conflicting reports on the genetic requirement for Nrp1 in zebrafish angiogenesis by demonstrating a fundamental role for NRP1 in mediating endogenous SEMA3A repulsion cues for ECs during physiological vascular morphogenesis in vivo*,* a function that is also conserved in humans. Furthermore, we found that NRP1 can further restrict angiogenesis by releasing sFLT1 in a growth factor-mediated negative feedback loop, unveiling a novel mechanism with potential clinical implication in preeclampsia pathophysiology.

## Supplementary Information

Below is the link to the electronic supplementary material.

Supplementary file1 (PDF 2236 kb)

Supplementary file 2 (MP4 8444 kb)

Supplementary file 3 (MP4 8424 kb)

Supplementary file 4 (MP4 8710 kb)

Supplementary file 5 (MP4 10464 kb)

Supplementary file 6 (MP4 28462kb)

Supplementary file 7 (MP4 29237kb)

## Data Availability

The authors declare that the data supporting the findings of this study are available within the paper and its Supplementary Information files. Should any raw data files be needed in another format they are available from the corresponding author upon reasonable request.

## References

[1] Potente M, Gerhardt H, Carmeliet P (2011) Basic and therapeutic aspects of angiogenesis. Cell 146(6):873–887 21925313 10.1016/j.cell.2011.08.039

[2] Hogan BM, Schulte-Merker S (2017) How to plumb a Pisces: understanding vascular development and disease using zebrafish embryos. Dev Cell 42(6):567–583 28950100 10.1016/j.devcel.2017.08.015

[3] Fantin A et al (2013) NRP1 acts cell autonomously in endothelium to promote tip cell function during sprouting angiogenesis. Blood 121(12):2352–2362 23315162 10.1182/blood-2012-05-424713 PMC3606070

[4] Raimondi C et al (2016) NRP1 function and targeting in neurovascular development and eye disease. Prog Retin Eye Res 52:64–83 26923176 10.1016/j.preteyeres.2016.02.003 PMC4854174

[5] Fantin A et al (2014) Neuropilin 1 (NRP1) hypomorphism combined with defective VEGF-A binding reveals novel roles for NRP1 in developmental and pathological angiogenesis. Development 141(3):556–562 24401374 10.1242/dev.103028 PMC3899814

[6] Gelfand MV et al (2014) Neuropilin-1 functions as a VEGFR2 co-receptor to guide developmental angiogenesis independent of ligand binding. Elife 3:e03720 25244320 10.7554/eLife.03720 PMC4197402

[7] Acevedo LM et al (2008) Semaphorin 3A suppresses VEGF-mediated angiogenesis yet acts as a vascular permeability factor. Blood 111(5):2674–2680 18180379 10.1182/blood-2007-08-110205 PMC2254547

[8] Casazza A et al (2011) Systemic and targeted delivery of semaphorin 3A inhibits tumor angiogenesis and progression in mouse tumor models. Arterioscler Thromb Vasc Biol 31(4):741–749 21205984 10.1161/ATVBAHA.110.211920

[9] Maione F et al (2009) Semaphorin 3A is an endogenous angiogenesis inhibitor that blocks tumor growth and normalizes tumor vasculature in transgenic mouse models. J Clin Invest 119(11):3356–3372 19809158 10.1172/JCI36308 PMC2769187

[10] Ochsenbein AM et al (2016) Endothelial cell-derived semaphorin 3A inhibits filopodia formation by blood vascular tip cells. Development 143(4):589–594 26884395 10.1242/dev.127670

[11] Vieira JM, Schwarz Q, Ruhrberg C (2007) Selective requirements for NRP1 ligands during neurovascular patterning. Development 134(10):1833–1843 17428830 10.1242/dev.002402 PMC2702678

[12] Gu C et al (2003) Neuropilin-1 conveys semaphorin and VEGF signaling during neural and cardiovascular development. Dev Cell 5(1):45–57 12852851 10.1016/s1534-5807(03)00169-2 PMC3918747

[13] Torres-Vazquez J et al (2004) Semaphorin-plexin signaling guides patterning of the developing vasculature. Dev Cell 7(1):117–123 15239959 10.1016/j.devcel.2004.06.008

[14] Zygmunt T et al (2011) Semaphorin-PlexinD1 signaling limits angiogenic potential via the VEGF decoy receptor sFlt1. Dev Cell 21(2):301–314 21802375 10.1016/j.devcel.2011.06.033 PMC3156278

[15] Kim J et al (2011) Semaphorin 3E-Plexin-D1 signaling regulates VEGF function in developmental angiogenesis via a feedback mechanism. Genes Dev 25(13):1399–1411 21724832 10.1101/gad.2042011 PMC3134083

[16] Fukushima Y et al (2011) Sema3E-PlexinD1 signaling selectively suppresses disoriented angiogenesis in ischemic retinopathy in mice. J Clin Invest 121(5):1974–1985 21505259 10.1172/JCI44900 PMC3083763

[17] Kok FO et al (2015) Reverse genetic screening reveals poor correlation between morpholino-induced and mutant phenotypes in zebrafish. Dev Cell 32(1):97–108 25533206 10.1016/j.devcel.2014.11.018 PMC4487878

[18] Lee P et al (2002) Neuropilin-1 is required for vascular development and is a mediator of VEGF-dependent angiogenesis in zebrafish. Proc Natl Acad Sci U S A 99(16):10470–10475 12142468 10.1073/pnas.162366299 PMC124944

[19] Martyn U, Schulte-Merker S (2004) Zebrafish neuropilins are differentially expressed and interact with vascular endothelial growth factor during embryonic vascular development. Dev Dyn 231(1):33–42 15305285 10.1002/dvdy.20048

[20] Wang L, Mukhopadhyay D, Xu X (2006) C terminus of RGS-GAIP-interacting protein conveys neuropilin-1-mediated signaling during angiogenesis. FASEB J 20(9):1513–1515 16754745 10.1096/fj.05-5504fje

[21] Hillman RT et al (2011) Neuropilins are positive regulators of Hedgehog signal transduction. Genes Dev 25(22):2333–2346 22051878 10.1101/gad.173054.111 PMC3222900

[22] Fantin A et al (2015) NRP1 regulates CDC42 activation to promote filopodia formation in endothelial tip cells. Cell Rep 11(10):1577–1590 26051942 10.1016/j.celrep.2015.05.018 PMC4528263

[23] Delcourt N et al (2015) Targeted identification of sialoglycoproteins in hypoxic endothelial cells and validation in zebrafish reveal roles for proteins in angiogenesis. J Biol Chem 290(6):3405–3417 25384978 10.1074/jbc.M114.618611 PMC4319010

[24] Dell AL et al (2013) cAMP-induced expression of neuropilin1 promotes retinal axon crossing in the zebrafish optic chiasm. J Neurosci 33(27):11076–11088 23825413 10.1523/JNEUROSCI.0197-13.2013 PMC3719991

[25] Lowe V et al (2019) Neuropilin 1 mediates epicardial activation and revascularization in the regenerating zebrafish heart. Development. 10.1242/dev.174482 31167777 10.1242/dev.174482 PMC6633600

[26] Taku AA et al (2016) Attractant and repellent cues cooperate in guiding a subset of olfactory sensory axons to a well-defined protoglomerular target. Development 143(1):123–132 26732841 10.1242/dev.127985 PMC4725209

[27] Yee CS et al (1999) Molecular cloning, expression, and activity of zebrafish semaphorin Z1a. Brain Res Bull 48(6):581–593 10386838 10.1016/s0361-9230(99)00038-6

[28] Shaw KM et al (2006) fused-somites-like mutants exhibit defects in trunk vessel patterning. Dev Dyn 235(7):1753–1760 16607654 10.1002/dvdy.20814

[29] Shoji W, Yee CS, Kuwada JY (1998) Zebrafish semaphorin Z1a collapses specific growth cones and alters their pathway in vivo. Development 125(7):1275–1283 9477326 10.1242/dev.125.7.1275

[30] Raimondi C et al (2014) Imatinib inhibits VEGF-independent angiogenesis by targeting neuropilin 1-dependent ABL1 activation in endothelial cells. J Exp Med 211(6):1167–1183 24863063 10.1084/jem.20132330 PMC4042645

[31] Hamm MJ, Kirchmaier BC, Herzog W (2016) Sema3d controls collective endothelial cell migration by distinct mechanisms via Nrp1 and PlxnD1. J Cell Biol 215(3):415–430 27799363 10.1083/jcb.201603100 PMC5100291

[32] Sztal TE, Stainier DYR (2020) Transcriptional adaptation: a mechanism underlying genetic robustness. Development. 10.1242/dev.186452 32816903 10.1242/dev.186452

[33] Aspalter IM et al (2015) Alk1 and Alk5 inhibition by Nrp1 controls vascular sprouting downstream of Notch. Nat Commun 6:7264 26081042 10.1038/ncomms8264 PMC4557308

[34] Gerhardt H et al (2004) Neuropilin-1 is required for endothelial tip cell guidance in the developing central nervous system. Dev Dyn 231(3):503–509 15376331 10.1002/dvdy.20148

[35] Schwarz Q et al (2009) Neuropilin 1 signaling guides neural crest cells to coordinate pathway choice with cell specification. Proc Natl Acad Sci U S A 106(15):6164–6169 19325129 10.1073/pnas.0811521106 PMC2661313

[36] Schwarz Q et al (2004) Vascular endothelial growth factor controls neuronal migration and cooperates with Sema3A to pattern distinct compartments of the facial nerve. Genes Dev 18(22):2822–2834 15545635 10.1101/gad.322904 PMC528901

[37] Serini G et al (2003) Class 3 semaphorins control vascular morphogenesis by inhibiting integrin function. Nature 424(6947):391–397 12879061 10.1038/nature01784

[38] Carrer A et al (2012) Neuropilin-1 identifies a subset of bone marrow Gr1- monocytes that can induce tumor vessel normalization and inhibit tumor growth. Cancer Res 72(24):6371–6381 23222303 10.1158/0008-5472.CAN-12-0762

[39] Reidy KJ et al (2009) Semaphorin3a regulates endothelial cell number and podocyte differentiation during glomerular development. Development 136(23):3979–3989 19906865 10.1242/dev.037267 PMC2778745

[40] Guttmann-Raviv N et al (2007) Semaphorin-3A and semaphorin-3F work together to repel endothelial cells and to inhibit their survival by induction of apoptosis. J Biol Chem 282(36):26294–26305 17569671 10.1074/jbc.M609711200

[41] Wu LF et al (2025) Semaphorin 3A protects against thoracic aortic aneurysm dissection by suppressing aortic angiogenesis. Angiogenesis 28(3):39 40580205 10.1007/s10456-025-09992-6

[42] Yu W et al (2013) Inhibition of pathological retinal neovascularization by semaphorin 3A. Mol Vis 19:1397–1405 23825919 PMC3695760

[43] Bouvree K et al (2012) Semaphorin3A, Neuropilin-1, and PlexinA1 are required for lymphatic valve formation. Circ Res 111(4):437–445 22723296 10.1161/CIRCRESAHA.112.269316 PMC3861899

[44] Lettieri A et al (2023) SEMA6A drives GnRH neuron-dependent puberty onset by tuning median eminence vascular permeability. Nat Commun 14(1):8097 38062045 10.1038/s41467-023-43820-z PMC10703890

[45] Matsuoka RL et al (2016) Radial glia regulate vascular patterning around the developing spinal cord. Elife. 10.7554/eLife.20253 27852438 10.7554/eLife.20253 PMC5123865

[46] Wild R et al (2017) Neuronal sFlt1 and Vegfaa determine venous sprouting and spinal cord vascularization. Nat Commun 8:13991 28071661 10.1038/ncomms13991 PMC5234075

[47] Moore KH et al (2019) sFlt-1 production in endothelial cells is regulated in part by VEGF receptor signaling. FASEB J 33(IssueS1):865.11

[48] Ahmad S et al (2011) Autocrine activity of soluble Flt-1 controls endothelial cell function and angiogenesis. Vasc Cell 3(1):15 21752276 10.1186/2045-824X-3-15 PMC3173355

[49] Fan X et al (2014) Endometrial VEGF induces placental sFLT1 and leads to pregnancy complications. J Clin Invest 124(11):4941–4952 25329693 10.1172/JCI76864 PMC4347223

[50] Habeck H et al (2002) Analysis of a zebrafish VEGF receptor mutant reveals specific disruption of angiogenesis. Curr Biol 12(16):1405–1412 12194822 10.1016/s0960-9822(02)01044-8

[51] Lange M et al (2022) Zebrafish mutants in vegfab can affect endothelial cell proliferation without altering ERK phosphorylation and are phenocopied by loss of PI3K signaling. Dev Biol 486:26–43 35337795 10.1016/j.ydbio.2022.03.006 PMC11238767

[52] Rossi A et al (2016) Regulation of Vegf signaling by natural and synthetic ligands. Blood 128(19):2359–2366 27557946 10.1182/blood-2016-04-711192 PMC5766839

