# Supplementary material for: Neuropilin 1 (NRP1) conveys SEMA3A signals to restrict physiological angiogenesis
Source: Angiogenesis. 2026 May 3;29(3):31. doi: 10.1007/s10456-026-10033-z (PMC13136202; doi:10.1007/s10456-026-10033-z)
Supplement: Supplementary file 1 — Supplementary file1 (PDF 2236 kb) [file 10456_2026_10033_MOESM1_ESM.pdf]

Neuropilin 1 (NRP1) conveys SEMA3A signals to restrict physiological angiogenesis

Marco Spreafico[1], Elena Guzzolino[1,2], Francesca Fanuele[1,3], Gaia Gestri[4], Carlotta Tacconi[1,5,6], Sara Palermo[1], Matilde Tricco[1], Valeria Catroppa[1], Ayazhan Aiypova[1], Laura Denti[7,8], Caroline Pellet-Many[9], Christiana Ruhrberg[7], Alessandro Fantin[1,*]

[1] University of Milan, Department of Biosciences, Via G. Celoria 26, 20133, Milan, Italy.
[2] Current affiliation: Institute of Clinical Physiology, National Research Council (IFC-CNR), Florence, Italy.
[3] Current affiliation: Adaptive Immunity Laboratory, Istituto di Ricovero e Cura a Carattere Scientifico (IRCCS) Humanitas Research Hospital, Rozzano, Italy.
[4] UCL Department of Cell and Developmental Biology, University College London, Gower Street, London WC1E 6BT, UK.
[5] Current affiliation: Università Vita-Salute San Raffaele, Via Olgettina, 58, 20132 Milano, Italy.
[6] Current affiliation: Institute of Clinical Physiology, National Research Council (IFC-CNR), Milan, Italy.
[7]UCL Institute of Ophthalmology, University College London, 11-43 Bath Street, London EC1V 9EL, UK.
[8] Current affiliation: The Francis Crick Institute, 1 Midland Rd, London, NW1 1AT, UK.
[9] Department of Comparative Biomedical Sciences, Royal Veterinary College, Royal College Street, London NW1 0TU, UK.

* Corresponding author:
Professor Alessandro Fantin, Department of Biosciences, University of Milan, Via G. Celoria 26, 20133, Milan, Italy; Email: alessandro.fantin@unimi.it; Tel: +39 02 503 14952

Methods

Zebrafish lines

Zebrafish (*Danio rerio*) were maintained according to international (EU Directive 2010/63/EU) and national guidelines (Italian decree No 26 of the 4th of March 2014, UK Home Office guidelines). *Tg(fli1a:EGFP)^{y1}* [1], *Tg(kdrl:EGFP)^{s843}* [2], *Tg(kdrl:Hsa.HRAS-mCherry)^{s896}* (abbreviated to *Tg(kdrl:mCherry)* in the main text) [3], *Tg(fli1:nlsEGFP)^{y7}*(abbreviated to *Tg(fli1:nEGFP)* in the main text) [4], *nrp1a^{sa1485}* (Zebrafish Mutation Project, Sanger Centre, Cambridge, UK) and *nrp1b^{fh278}* (Zebrafish Tilling Project) zebrafish strains were used. Embryos were staged as described in Kimmel et al. [5] and raised in fish water (0.01%Instant Ocean, 0.01% sodium bicarbonate, 0.019% calcium sulphate, 0.1% Methylene Blue) at 28 °C in Petri dishes, according to established techniques. Embryonic ages are expressed in hours post fertilization (hpf) and days post fertilization (dpf). Embryos were anesthetized with 0.016% tricaine (Ethyl 3-aminobenzoate methanesulfonate salt, Sigma-Aldrich) before proceeding with experimental protocols. When needed, 0.003% 1-phenyl-2-thiourea (PTU, Sigma-Aldrich) was added to fish water to prevent pigmentation. For imaging purpose, embryos were fixed in 4% PFA in PBS overnight at 4°C and then they were dehydrated by methanol scale.

Genotyping

To assess the genotype of *nrp1a^{sa1485}* and *nrp1b^{fh278}* adult fish, tail fin clipping was performed. Fin clips were digested in lysis buffer (5mM Tris-HCl pH 7.5, 1mM EDTA pH 8.0, 0.65 Tween-20, 0.6% glycerol in H_2O) with 1 mg/ml Proteinase K (Sigma-Aldrich) at 55°C for 4 hours. To assess the genotype of *nrp1a^{sa1485}* and *nrp1b^{fh278}* embryos, either whole 2 dpf embryos or 5 dpf embryo tails

were lysed with 20 µl of G1 buffer (25 mM NaOH, 0.2 mM EDTA) at 98°C for 15 minutes and then lysis was stopped by adding 20 µl of G2 buffer (40 mM Tris-HCl pH 5.5) The genotype of *nrp1a*^{sa1485} fish was assessed by KASP (Kompetitive Allele Specific PCR) assay, using the KASP TF V4.0 2X Mastermix Std ROX TF kit (LGC Genomics) with specific *nrp1a* primers and probes (KBD Assay, LGC genomics) according to manufacturer's instructions on a CFX Connect Real-Time PCR Detection System (Bio-Rad). The genotype of *nrp1b*^{fh278} fish was assessed by PCR using the following primers: *nrp1b* wt FW 5'-AATAGGAGTATCCACATGCTGCTTATAA-3'; *nrp1b* wt REV 5'-GTAGTCGGAGGTGAATTTGATGACTA-3'; *nrp1b* mut FW 5'-CTCCGATTATCTCTAGTGGAAACTCATA-3'; *nrp1b* mut REV 5'-TTAAAGACCATATGTTTGTCTTTGTGTG-3'. 10 µl reactions were assembled including 0.1 µM *nrp1b* wt FW, 0.1 µM *nrp1b* wt REV, 0.5 µM *nrp1b* mut FW, 0.05 µM *nrp1b* mut REV and 2 µl of DNA using the WonderTaq kit (EuroClone) according to manufacturer's instruction. PCR was performed for 40 cycles with T_{ann} at 51°C and 20 seconds extension time. PCR products were run on a 2.5% agarose gel.

Morpholino (MO) injections

Tg(kdrl:EGFP) zebrafish embryos were injected at 1-4 cell stage with *nrp1a(/b)* translation blocking MO 5'-GAATCCTGGAGTTCGGAGTGCGGAA-3' (*nrp1a(/b)*-MO, Gene Tools, previously published as a *nrp1a*-MO) [6, 7], *nrp1b* splicing blocking MO 5'-TATACCTGTACGGTGTATCTCATAG-3' (*nrp1b*-MO, Gene Tools) [8] and *sema3ab* splicing MO 5'-AAATGTGTCTTACCGTTGAGCCATC-3'MO (*sema3ab*-MO, Gene Tools) [9], either alone or in combination, by using a PV850 microinjector (World Precision Instrument). An equal concentration of standard control MO 5'-CCTCTTACCTCAGTTACAATTTATA-3' (Std-MO, Gene Tools) was injected in control embryos.

Embryo transplant

Cells from *Tg(fli1a:EGFP)* embryos injected with either Std-MO or 0.6 pmol/e of *nrp1a(/b)* MO were collected from the donor's animal pole and transplanted into the host's lateral margin zone of blastula-stage *Tg(kdrl:mCherry)* embryos, as previously done [10], and analysed at 36 hpf.

Cell culture and transfection

Cells were grown at 37°C in a humidified 5% CO_2 incubator. HEK-293T cells were cultured in DMEM (EuroClone) supplemented with 10% fetal bovine serum (FBS, EuroClone), 1% L-glutammine (EuroClone) and 1% Antibiotic-Antimycotic solution (EuroClone). 120,000 HEK were seeded in 6-well plates and the following day they were transfected with 2.5 µg of plasmid encoding either AP (Alkaline Phosphatase) only or SEMA3A-AP [11] using Lipofectamine 2000 (Invitrogen) according to the manufacturer's instructions. Transfected HEKs were grown for three days and then they were selected by a three day-treatment with 1 mg/ml Zeocin (Invitrogen). HUVECs were cultured in EGM2 (endothelial growth medium) with supplements (Promocell) in plates coated with 10 µg/ml human plasma fibronectin (Merck Millipore). 120,000 HUVECs were seeded in 6-well plates and the following day they were transfected with 30 nM SMARTpool siRNA targeting *NRP1* (Dharmacon) or *Silencer*® negative control siRNA (Applied Biosystems) using Lipofectamine RNAIMAX (Invitrogen), according's to manufacturer instructions, in EGM2 (Lonza). For RNA extraction, 70,000 transfected HUVECs were seeded in 12-well plates and let grow overnight before proceeding. For proliferation assay, 8,000 transfected HUVECs were seeded in 48-well plates.

Co-culture

40,000 transfected HUVECs were plated on 13mm cover glasses in 24-well plates the day after transfection. At 48 hours after HUVEC transfection, 40,000 zeocin-selected HEKs were added

to HUVECs in each well. Co-culture was maintained for 24 hours in EGM2 with supplements. In each independent experiment, each condition was performed in triplicate. For laser scanning confocal imaging, cells were fixed in 4% PFA in PBS for 10 minutes at room temperature. For live imaging experiments, green fluorescent protein (GFP)-expressing HUVECs were used (Angio-Proteomie) and both HUVECs and HEKs were incubated 10 minutes at 37°C with 5 µg/ml Hoechst 33342 (Merck) before live imaging.

SEMA3A treatment

8,000 HUVECs in 48-well plates or 35,000 HUVECs in 12-well plates were seeded for proliferation assay and RNA extraction, respectively. The following day medium was replaced and 500 ng/ml human recombinant furin-activated SEMA3A (PeproTech) was added to the culture. For RNA extraction purpose, cells were cultured in 30% EGM2 with supplements and 70% EBM2 (endothelial basal medium, not including supplements) for 24 hours. For proliferation assays cells were cultured in EGM2. 0.1% BSA in PBS was used as a control.

Proliferation assay

HUVEC proliferation was assessed by Crystal violet staining. SEMA3A-treated cells after 72 hours of treatment or siRNA-transfected cells at 48 and 96 hours post transfection were fixed with 70% cold EtOH at 4°C for 20 min. Cells were then stained with 0.2% crystal violet in EtOH at RT for 30 minutes shaking. After 4 PBS washes plates were let dry overnight. The following day 33% acetic acid was added to each well for 30 minutes at RT shaking to solubilize the staining. Crystal violet absorbance was then read using an Ensight Multimode Plate Reader (PerkinElmer).

RT-qPCR

Total RNA was extracted from 20 pooled zebrafish embryos at 2 dpf by using TRIzol reagent (Invitrogen) followed by purification with Total RNA Miniprep kit (Monarch), according to the manufacturer's instructions, or from HUVECs cultured in 12-well plates by directly using the Total RNA Miniprep kit (Monarch). 1 µg total RNA was used to perform reverse transcription (RT) reaction with the High-Capacity cDNA Reverse Transcription kit (Applied Biosystems). 10 µl qPCR reactions were prepared with 2 µl of a 1:10 dilution of RT products and 0.25 µM primers and performed using the Luna Universal qPCR Master Mix (New England Biolabs), according to the manufacturer's instructions, on a CFX Connect Real-Time PCR Detection system (BioRad). The following primers were used: zebrafish soluble *flt1* (*sflt1*) FW 5'-ACCACCTCAAATCCAATGGCT-3' and s*flt1* REV 5'-TGGCTGAGATAAGAGTGCTGC-3'; zebrafish membrane-bound *flt1* (*mflt1*) FW 5'-GCTCGGGATTCAGGAACGTA-3' and *mflt1* REV 5'-CCATGTTATGTGTGGCTGTGG-3'; zebrafish *eef1a1l1* FW 5'-CTGGAGGCCAGCTCAAACAT-3' and *eef1a1l1* REV 5'-ATCAAGAAGAGTAGTACCGCTAGCAT-3'; human *sFLT1* FW 5'-GTTGGGACTGTGGGAAGAAAC-3' and *sFLT1* REV 5'-GGAGATCCGAGAGAAAACAGC-3'; human *mFLT1* FW 5'-GCACTACACATGGAGCCTAAGA-3' and *mFLT1* REV 5'-CCGTCAGAATCCTCCTCTTCC-3'; human *NRP1* FW 5'-ATGGATTCCCTGATGTTGG-3' and *NRP1* REV 5'-TGTAGTTTGCTGAGAAACCT-3'; human *RPLP0* FW 5'-CAGATTGGCTACCCAACTGTT-3' and *RPLP0* REV 5'-GGGAAGGTGTAATCCGTCTCC-3'.

Whole mount *in situ* hybridization (WISH)

To synthetize *sema3aa* and *sema3ab* probes, RT was performed as described in the RT-qPCR section and cDNA was amplified by PCR reaction using the following primers: *sema3aa* FW 5'-CAGATTTAGGGACCGTTCTG-3'; *sema3aa*-T7 REV: 5'-CGTAATACGACTCACTATAGGGGCTTCTTCTCGCTTCCAA-3'; *sema3ab* FW 5'-

CTACAACACCACGATGAGGC-3'; *sema3ab*-T7 REV: 5'-CGTAATACGACTCACTATAGGGGATCTGGCTGGTGTGTGATT-3'. PCR reactions were performed using the OneTaq® Quick-Load® 2X Master Mix with Standard Buffer (New England Biolabs) and 2 µl of a 1:10 dilution of RT products. PCRs were conducted setting 35 cycles with T_{ann} at 54 and 55°C for *sema3aa* and *sema3ab*, respectively. Probes were synthetized using T7 RNA polymerases kit (Promega) and digoxigenin (DIG) RNA labelling mix (Roche). WISH was carried out as previously described (https://zfin.atlassian.net/wiki/spaces/prot/pages/369265015/Thisse+Lab+-+In+Situ+Hybridization+Protocol+-+2010+update). Digital images of all embryos were captured using a stereomicroscope equipped with digital camera with LAS Leica Imaging software (Leica). Some embryos were cryosectioned following WISH and imaged with an Olympus CKX53 microscope equipped with an Olympus EP50 camera using a 20X objective.

Western blotting

To extract total protein from zebrafish embryos, at least 30 whole embryos at 2 dpf or heads from 5 dpf larvae were pooled and suspended in deyolking buffer (0.5x Ginzburg Fish Ringer: 55mM NaCl, 1.8 mM KCl, 1.25 mM $NaHCO_3$, 1.25 mM $CaCl_2$) to remove yolk sac, washed with PBS and lysed with RIPA buffer (50 mM Tris-HCl pH 8.0, 150 mM NaCl, 0.1% SDS, 0.5% sodium deoxycholate, 1% TRITON X-100). Samples were sonicated, centrifuged at 4°C for 10 minutes at 16,000 g and then supernatants were collected. To extract protein from HUVECs, cells were lysed with RIPA buffer, incubated on ice for 30 minutes, centrifuged at 4°C for 10 minutes at 16,000 g and then supernatants were collected. Total proteins were quantified using the Quantum Protein BCA protein assay kit (Euroclone). 10 µg of total protein were heat-denatured and separated by SDS-PAGE using a Novex 4-12% Tris-Glycine Plus Wedge Well gel (Invitrogen) and then blotted on a nitrocellulose membrane by using the iBlot2 NC Regular Stack kit (Invitrogen) and an iBlot2 Dry Blotting System (Invitrogen). Membranes were blocked in 5% milk PBS-T (0.1% TWEEN-20 in PBS) for 1 hour at room temperature and incubated with primary antibody diluted in 5% BSA PBS-T overnight at 4°C. Membranes were then incubated with secondary antibody diluted in 5% milk PBS-T for 1 hour at room temperature. Protein bands were detected by using LiteAblot ECL kits (Euroclone). Primary antibodies were rabbit anti-NRP1 1:1000 (Abcam EPR3113), rabbit anti-αβ-tubulin 1:1000 (Cell Signaling Technology #2148) and mouse anti-β-actin 1:1000 (Cell Signaling Technology #3700). Secondary antibodies were goat anti-rabbit HRP-linked (Cell Signaling Technology #7074) and horse anti-mouse HRP-linked (Cell Signaling Technologies #7076).

Immunofluorescence

For zebrafish immunofluorescence, methanol-stored 28 hpf *Tg(kdrl:Hsa.HRAS-mCherry)*;*Tg(fli1:nlsEGFP)* zebrafish embryos were rehydrated, permeabilized with 10 µg/ml Proteinase K for 20 minutes, post-fixed with 4% PFA in PBS for 20 minutes at room temperature, then incubated in blocking solution (10% donkey serum, 1% DMSO, 0.5% Triton X-100 in PBS) for 1 hour at room temperature. Embryos were then incubated with rabbit anti-phospho histone H3 (Ser10) antibody 1:400 (Merck Millipore, 06-570) in blocking solution overnight at 4°C. The following day embryos were incubated with Cy5 donkey anti-rabbit antibody 1:500 (Jackson ImmunoResearch, 711-175-152) in blocking solution overnight at 4°C.

For cell immunofluorescence, fixed cells were incubated in blocking solution (10% donkey serum, 0.1% Triton X-100 in PBS) for 1 hour at room temperature, then incubated overnight at 4°C with mouse anti-PECAM1 1:50 (Biosite, JC/70A) primary antibody diluted in blocking solution. The following day cells were incubated with FITC donkey anti-mouse 1:200 (Jackson ImmunoResearch 715-096-150) secondary antibody together with phalloidin-TRITC 1:400 (Sigma-Aldrich) diluted in blocking solution for 2 hours at room temperature, then incubated in 0.5 µg/ml DAPI stain (Cell Signaling Technology) for 10 minutes at room temperature.

Confocal imaging

For zebrafish experiments, embryos were mounted in 1% low-melting agarose in PBS. Images were acquired using an ECLIPSE Ti2-E AX R laser scanning confocal microscope (Nikon). For live imaging experiments, 26 hpf zebrafish embryos were mounted in 1% low-melting agarose in 0.016% tricaine fish water and incubated at 27°C o/n. Images were taken every 20 minutes by using an ECLIPSE Ti2-E with Yokogawa W1-SoRa spinning disk confocal microscope (Nikon) and Imaris (Oxford Instruments) was then used for image processing and movie generation.
For cell imaging, cells were mounted on microscope slides using Mowiol (Sigma-Aldrich) and three images per replicate were acquired using an ECLIPSE Ti2-E AX R laser scanning confocal microscope (Nikon). For live cell imaging experiments, cells were plated in 24-well plates and images were taken every 15 minutes by using an ECLIPSE Ti2-E with Yokogawa W1-SoRa spinning disk confocal microscope (Nikon) and Fiji software (https://imagej.net/software/fiji/) was then used for image processing and movie generation.

Image analysis

sema3aa and *sema3ab* expression in the dorsal and ventral myotomes of the same embryo was performed by measuring pixel mean grey values in regions-of-interest (ROIs) of the same size with Fiji after transforming WISH captures in black-and-white inverted images.
Gap areas in cell culture experiments were quantified with Fiji by selecting the PECAM1 channel (green) for each image and measuring the area not covered by HUVECs with the “Analyze particles” tool by setting 2000-infinity as size range (**Fig. S7a**). Total HUVEC area and HUVEC nuclei were quantified with Fiji by thresholding the PECAM1 signal for each image to measure the area in pixels covered by HUVECs and to build a mask to select DAPI-positive nuclei belonging to HUVECs only; HUVECs nuclei were then quantified with the “Analyze particles” tool (**Fig. S7b**).

Statistical analysis

Statistical analyses were performed with Prism 7 (GraphPad Software). To determine if two data sets were significantly different, we first assessed data Gaussian distribution by Shapiro–Wilk normality test. Then, when comparing two datasets, we used unpaired or paired two-tailed Student’s t test for normally distributed samples and Mann-Whitney test when samples were not normally distributed. When comparing more than two datasets without a parametric distribution, Kruskal–Wallis followed by Dunn’s multiple comparisons test was used, whereas 2-way ANOVA was used if including 2 independent variables. A p value < 0.05 was considered significant. In each graph, error bars represent the standard deviation of the mean (SD), as indicated in each figure legend together with the statistical test used, the p value range and sample numerosity.

Supplementary Figures

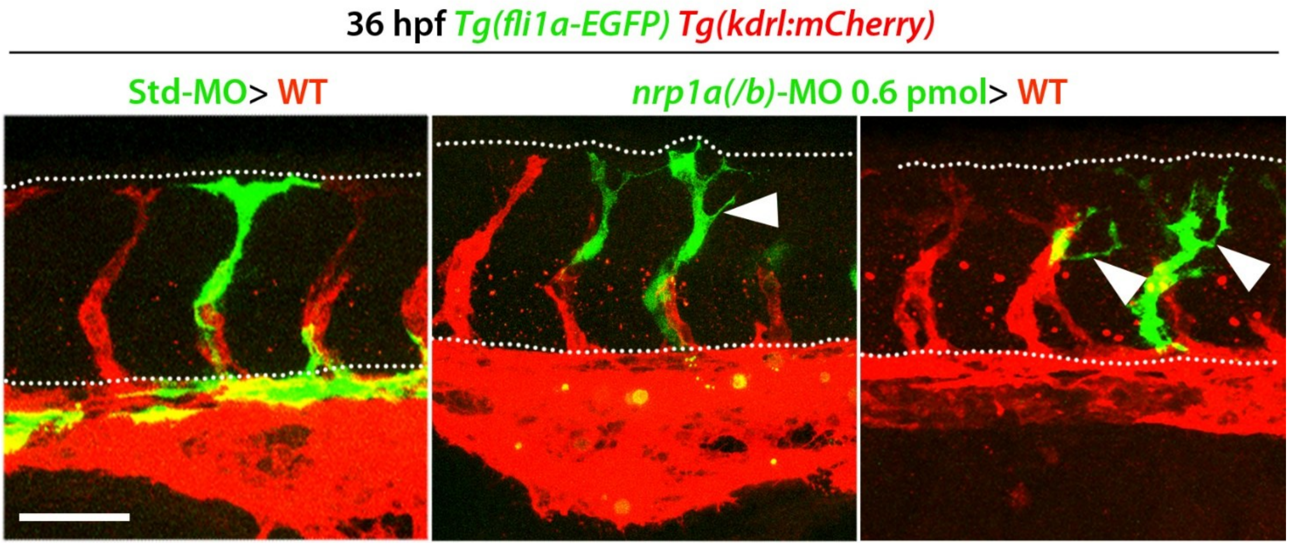

Figure S1. Mosaic targeting of *nrp1a(/b)* results in ISVs that reach the dorsal side of the trunk but ectopically sprout across the somite region.

Analysis of chimeric *Tg(fli1a:EGFP);Tg(kdrl:mCherry)* zebrafish embryos generated by transplanting cells from *Tg*(*fli1a:EGFP*) donor embryos injected with Std-MO (left panel) or 0.6 pmol/e of *nrp1a(/b)*-MO (central and right panels) into *Tg*(*kdrl:mCherry*) host embryos. Representative maximum-intensity projections of confocal z stacks through embryo trunks at 36 hpf; arrowheads indicate ectopic sprouts in the somites. Scale bar: 50 µm.

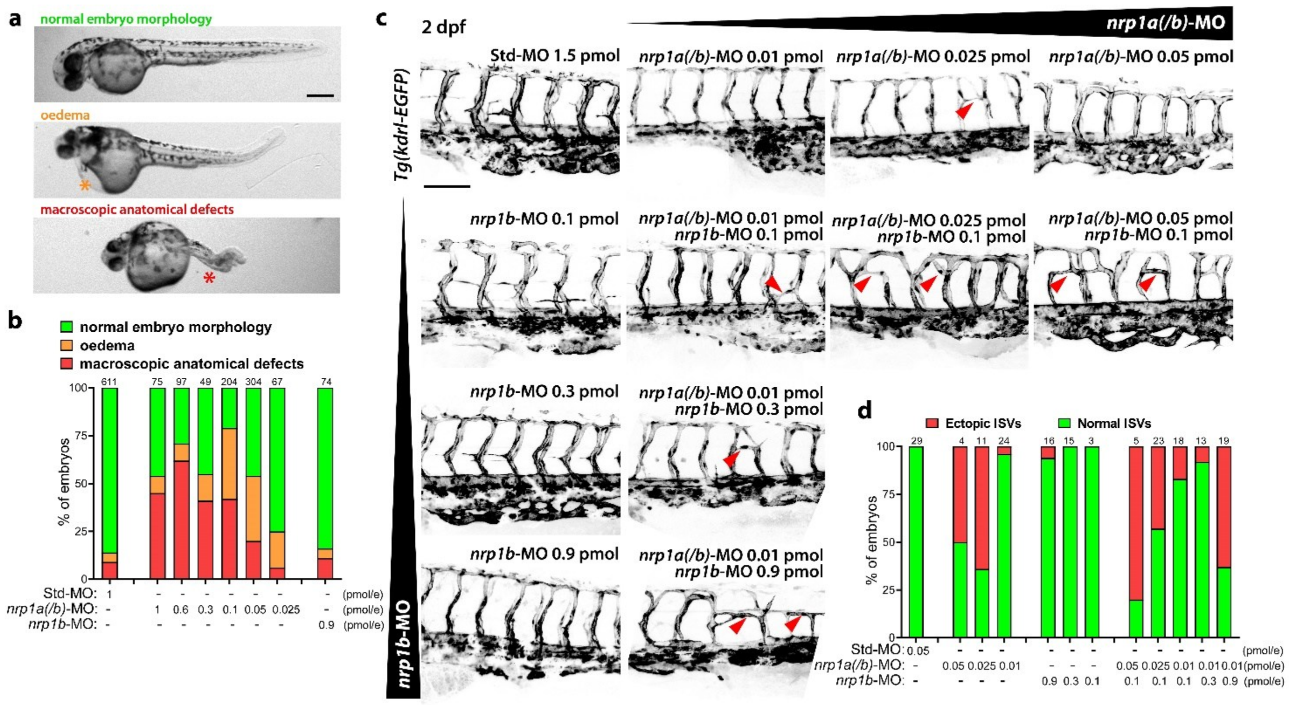

Figure S2. Morpholino dose titration based on macroscopic morphological defects and the presence of ectopic ISVs.

(**a-d**) Analysis of *Tg(kdrl:EGFP)* zebrafish embryos that were injected at 1-4 cell stage with control standard (Std)-MO versus *nrp1a(/b)*-MO, *nrp1b*-MO and combined *nrp1a(/b)*-MO*/nrp1b*-MO at the indicated doses. (**a**) Representative pictures of zebrafish embryos with normal morphology, oedema or macroscopic anatomical defects. (**b**) Scoring for unspecific MO toxicity. (**c**) Representative maximum-intensity projections of confocal z stacks through embryo trunks at 2 dpf. (**d**) Quantification of ectopic ISV penetrance. Scale bars: 250 µm in (**a**), 100 µm in (**c**). Numbers above histograms in (**b**) and (**d**) indicate the sample size of each group; e, embryo.

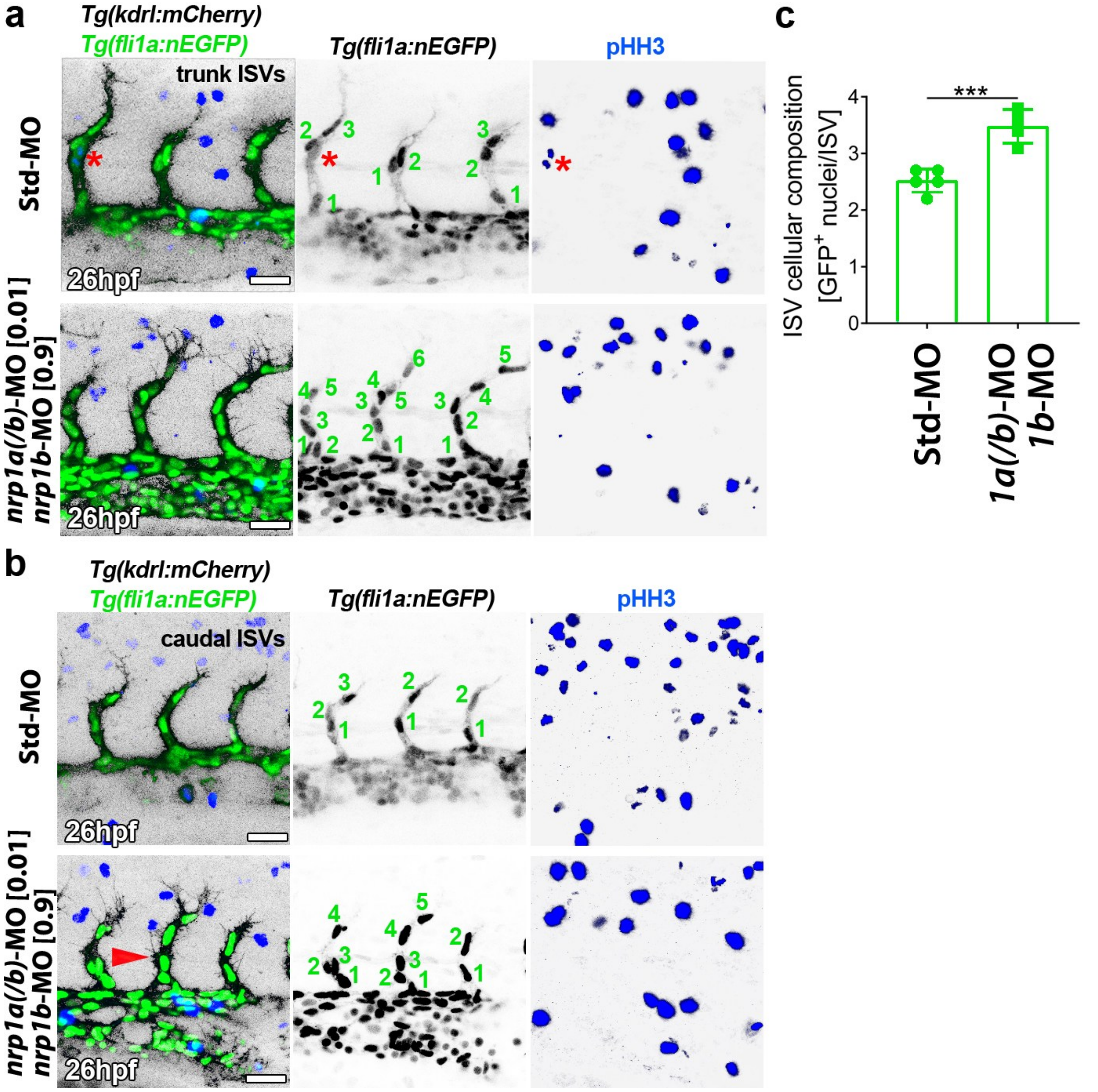

Figure S3. Nrp1 knockdown results in increased EC number within each ISV.

(**a-c**) Trunk vascularisation analysis of *Tg(kdrl:mCherry);Tg(fli1a:nEGFP)* zebrafish embryos that were injected at 1-4 cell stage with control standard (Std)-MO versus combined *nrp1a(/b)*-MO/*nrp1b*-MO at the indicated doses. Representative maximum-intensity projections of confocal z stacks through the central (**a**) or caudal (**b**) part of the embryo trunks at 26 hpf following immunostaining for pHH3 (blue channel); red arrowhead and asterisk indicate an example of ectopic ISV sprout and EC mitosis, respectively; scale bars: 20 µm. (**c**) Quantification of EC number in all fully extended ISVs in each embryo trunk; graph shows mean ± SD; each data point represents the value of a single embryo (n ≥ 4); ***, P < 0.001, unpaired Student-t test.

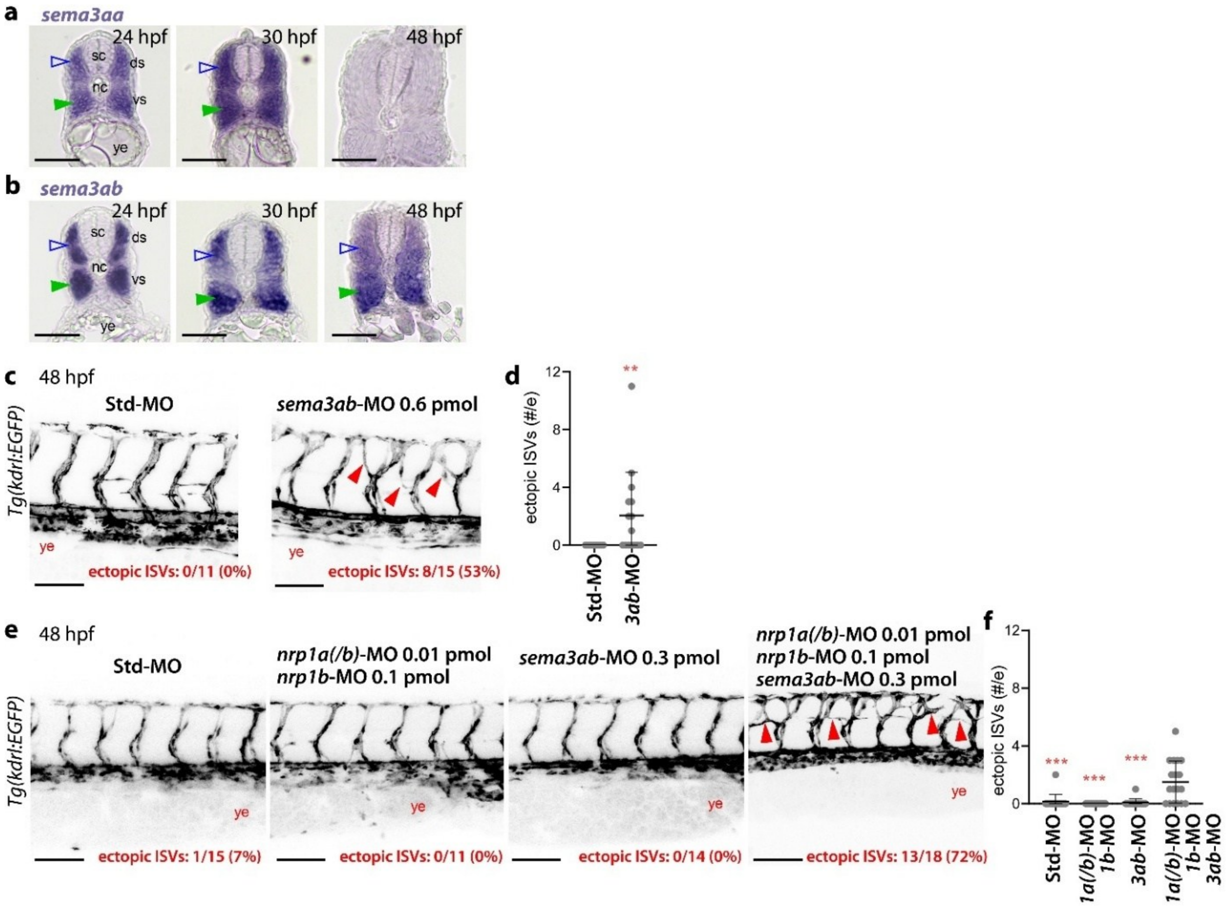

Figure S4. Nrp1 and Sema3a cooperate to prevent ectopic ISV sprouting across the somite region.

(**a,b**) Time course analysis of Sema3a expression in zebrafish embryos during trunk vascularisation. Representative pictures of 20 µm cryosections through zebrafish embryos at 24, 30 and 48 hpf following whole mount *in situ* hybridisation using antisense probes for *sema3aa* (**a**) and *sema3ab* (**b**); green full arrowheads and blue contour arrowheads indicate expression of transcripts for Sema3a ligands in the ventral and dorsal somites, respectively; ds, dorsal somite; nc, notochord; sc, spinal cord; vs, ventral somite; ye, yolk extension.

(**c,d**) Analysis of *Tg(kdrl:EGFP)* zebrafish that were injected at 1-4 cell stage with control Std-MO versus *sema3ab*-MO at the indicated dose. (**c**) Representative maximum-intensity projections of confocal z stacks through embryo trunks at 2 dpf; the frequency of embryos displaying the ectopic ISV phenotype is indicated in red in each confocal image; red arrowheads indicate examples of ectopic ISVs; ye, yolk extension. (**d**) Quantification of ectopic ISVs; each data point represents the value of a single embryo; n ≥ 11 embryos.

(**e,f**) Trunk vascularisation analysis of *Tg(kdrl:EGFP)* zebrafish embryos that were injected at 1-4 cell stage with control standard (Std)-MO versus double *nrp1a(/b)*-MO/*nrp1b*-MO, single *sema3ab*-MO and triple *nrp1a(/b)*-MO/*nrp1b*-MO/*sema3ab*-MO at the indicated doses. (**e**) Representative maximum-intensity projections of confocal z stacks through embryo trunks at 2 dpf; the frequency of embryos displaying the ectopic ISV phenotype is indicated in red in each confocal image; red arrowheads indicate examples of ectopic ISVs; ye, yolk extension. (**f**) Quantification of ectopic ISVs; each data point represents the value of a single embryo (n ≥ 11).

Scale bars: 50 µm (**a,b**); 100 µm (**c,e**). In (**d,f**), graphs show mean ± SD; **, P < 0.01; ***, P < 0.001, Mann-Whitney test (**d**) or Kruskal-Wallis test (versus triple MO injection, **f**).

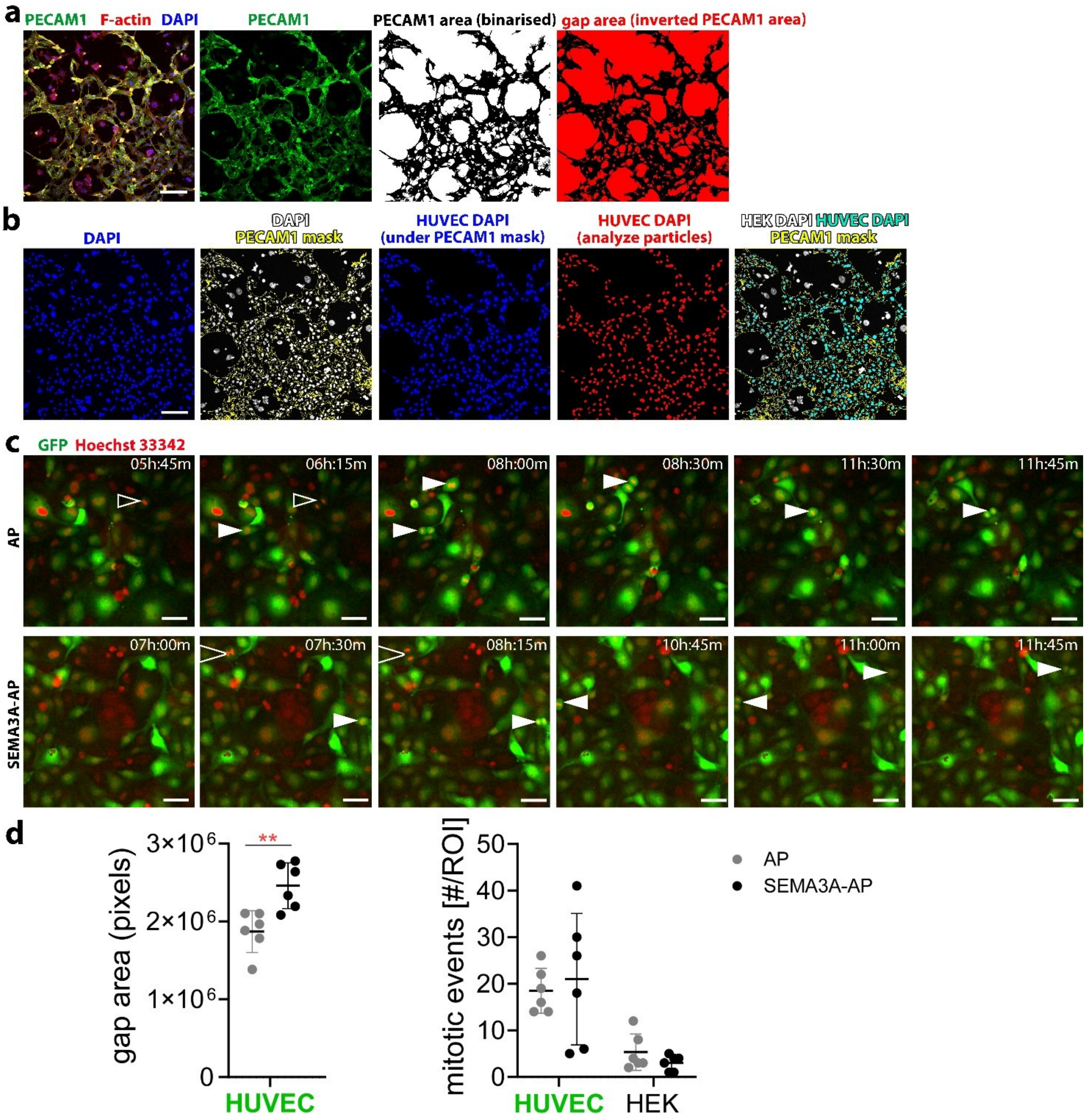

Figure S5. Characterization of the *in vitro* repulsion assay obtained by coculturing HUVECs with AP (alkaline phosphatase) only- or SEMA3A-AP-expressing HEK 293T cells.

(**a,b**) Representative sequential steps for image analysis to quantify gap areas in the EC monolayer (shown in red, **a**), and the total HUVEC area (shown in yellow, **b**) to be divided for the number of HUVECs (shown in red, **b**) to obtain the HUVEC single cell area.

(**c-d**) Time-lapse imaging of GFP-expressing HUVECs cocultured with AP- (top panels) or SEMA3A-AP-expressing HEKs (bottom panels) for 15 hours. (**c**) Representative confocal images at the indicated time points following staining of both cell types with Hoechst 33342 to label nuclei; white arrowheads indicate mitotic events in GFP-positive HUVECs, empty arrowheads indicate HEK cells undergoing mitosis. (**d**) Quantification of mitotic events in both cell types; graph shows mean ± SD; Each data point represents the value of a single well from one representative experiment out of 2 independent experiments (n = 6 wells).

Scale bars: 100 µm (**a,b**); 50 µm (**c**).

Movie M1.

Representative time-lapse movie assembled from maximum-intensity projections of confocal z stacks from *Tg(kdrl:mCherry);nrp1a*$^{-/-}$*;nrp1b*$^{+/-}$ zebrafish trunks starting from 26 hpf.

Movie M2.

Representative time-lapse movie assembled from maximum-intensity projections of confocal z stacks from *Tg(kdrl:mCherry);nrp1a*$^{-/-}$*;nrp1b*$^{-/-}$ zebrafish trunks starting from 26 hpf.

Movie M3.

Representative time-lapse movie assembled from maximum-intensity projections of confocal z stacks from *Tg(kdrl:mCherry);Tg(fli1a:nEGFP);nrp1a*$^{+/-}$*;nrp1b*$^{+/-}$ zebrafish trunks starting from 26 hpf.

Movie M4.

Representative time-lapse movie assembled from maximum-intensity projections of confocal z stacks from *Tg(kdrl:mCherry);Tg(fli1a:nEGFP);nrp1a*$^{-/-}$*;nrp1b*$^{-/-}$ zebrafish trunks starting from 26 hpf.

Movie M5.

Representative time-lapse movie assembled from confocal imaging of GFP-expressing HUVECs cocultured with AP-expressing HEKs for 15 hours, following staining of both cell types with Hoechst 33342 to label nuclei. White arrowheads indicate mitotic events in GFP-positive HUVECs, empty arrowheads indicate HEK cells undergoing mitosis.

Movie M6.

Representative time-lapse movie assembled from confocal imaging of GFP-expressing HUVECs cocultured with SEMA3A-AP-expressing HEKs for 15 hours, following staining of both cell types with Hoechst 33342 to label nuclei. White arrowheads indicate mitotic events in GFP-positive HUVECs, empty arrowheads indicate HEK cells undergoing mitosis.

References

1. Lawson, N.D. and B.M. Weinstein, *In vivo imaging of embryonic vascular development using transgenic zebrafish.* Dev Biol, 2002. **248**(2): p. 307-18.
2. Jin, S.W., et al., *Cellular and molecular analyses of vascular tube and lumen formation in zebrafish.* Development, 2005. **132**(23): p. 5199-209.
3. Chi, N.C., et al., *Foxn4 directly regulates tbx2b expression and atrioventricular canal formation.* Genes Dev, 2008. **22**(6): p. 734-9.
4. Roman, B.L., et al., *Disruption of acvrl1 increases endothelial cell number in zebrafish cranial vessels.* Development, 2002. **129**(12): p. 3009-19.
5. Kimmel, C.B., et al., *Stages of embryonic development of the zebrafish.* Dev Dyn, 1995. **203**(3): p. 253-310.
6. Lee, P., et al., *Neuropilin-1 is required for vascular development and is a mediator of VEGF-dependent angiogenesis in zebrafish.* Proc Natl Acad Sci U S A, 2002. **99**(16): p. 10470-5.
7. Hillman, R.T., et al., *Neuropilins are positive regulators of Hedgehog signal transduction.* Genes Dev, 2011. **25**(22): p. 2333-46.
8. Wang, L., D. Mukhopadhyay, and X. Xu, *C terminus of RGS-GAIP-interacting protein conveys neuropilin-1-mediated signaling during angiogenesis.* FASEB J, 2006. **20**(9): p. 1513-5.
9. Torres-Vazquez, J., et al., *Semaphorin-plexin signaling guides patterning of the developing vasculature.* Dev Cell, 2004. **7**(1): p. 117-23.
10. Cavodeassi, F., et al., *Early stages of zebrafish eye formation require the coordinated activity of Wnt11, Fz5, and the Wnt/beta-catenin pathway.* Neuron, 2005. **47**(1): p. 43-56.
11. Vieira, J.M., Q. Schwarz, and C. Ruhrberg, *Selective requirements for NRP1 ligands during neurovascular patterning.* Development, 2007. **134**(10): p. 1833-43.
